# A Causal Relationship Between Boron Seeds and the Selectivity and Growth Mode of Boron Nitride Nanotubes in Inductively‐Coupled Plasma

**DOI:** 10.1002/smll.202513512

**Published:** 2026-01-07

**Authors:** Aqeel. Alrebh, Liliana Gaburici, Dean Ruth, Mark Plunkett, Christopher T. Kingston, Keun Su Kim

**Affiliations:** ^1^ Quantum and Nanotechnologies Research Centre National Research Council Ottawa Ontario Canada; ^2^ Department of Mechanical and Industrial Engineering University of Toronto Toronto Ontario Canada

**Keywords:** BNNT selectivity, boron nitride nanotubes, boron seeds, growth mode, induction thermal plasma

## Abstract

We investigate a relationship between the size and abundance of boron (B) seeds and the selectivity of BNNTs in an inductively‐coupled plasma. Key parameters influencing the size and abundance of B seeds are discussed in relation to their impact on the selectivity of BNNTs versus other BN structures. Our statistical analysis suggests that the amount of BNNTs in the as‐produced material scales with B seed abundance. B partial pressure, adjusted through feedstock's feed rate, controls the seeds' abundance while maintaining their size range. The turbulence intensity and residence time in the B droplet formation zone, adjusted through the plasma power, influence both the size and abundance of seeds. Higher turbulence and longer residence times generally lead to larger seeds and broader size distributions. These two variables affect the cooling rate in the B seed‐BNNT formation zone, which inversely correlates with the seed size. Notably, BNNT diameters are consistent across all the tested conditions despite the seed size differences. We attribute this consistency to the growth mode, which is a hybrid of tangential and perpendicular modes, giving the seed‐tube structure a golf club‐like appearance. The perpendicular component is speculated to minimize the dependence of the nanotube diameter on the seed diameter.

## Graphical abstract

Boron seed formation governs BNNT selectivity in an inductively‐coupled plasma system. Local thermodynamics and flow conditions control the abundance and the size of these seeds. BNNT selectivity scales with seed abundance, while the nanotube diameters remain largely unaffected despite seed size changes. The latter is attributed to the hybrid tangential‐perpendicular growth mode that features a golf club‐like structure.

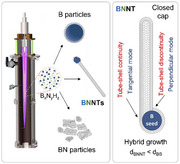

## 1 Introduction

Boron nitride nanotubes (BNNTs), a structural equivalent of carbon nanotubes (CNTs) consisting of boron (B) and nitrogen (N) in lieu of carbon, have become a subject of intensive research within the domains of synthesis, purification, and applications. BNNTs have *sp^2^*‐hybridized B‐N bonding of a polar nature, exhibiting distinct physical and chemical characteristics such as a wide band gap upward of 5.5 eV nearly independent of nanotube diameter, number of walls, and chirality [1]. Their high Young's modulus and tensile strength [2], dimension‐dependent high thermal conductivity [3, 4, 5], and high chemical stability and resistance to thermal oxidation [6, 7] place this material in a position to enhance engineering functionalities of various nanocomposites, adhesives, and 3D‐printed microarchitectures [8, 9, 10, 11, 12, 13, 14, 15, 16]. BNNTs display a piezoelectric character which has been leveraged in producing stretchable vibration sensors and energy nanogenerators [17, 18]. Additionally, boron‐10‐derived BNNTs show high neutron radiation shielding and sensing capabilities due to the high neutron absorption cross‐section of boron‐10 [19, 20, 21]. Also, BNNTs have been shown to exhibit superhydrophobic behavior, and thus are adequate for dry and self‐cleaning, and anticorrosive coatings [22, 23]. Several research efforts have demonstrated the biocompatibility of functionalized and non‐functionalized BNNTs with proteins and cells, and thus have been explored in various biological and pre‐clinical environments, including anticancer drug delivery and nanomedicine [24, 25, 26, 27, 28, 29]. Such applications mandate a synthesis method where BNNT selectivity is high compared to by‐products.

Following the proposition of BNNTs stability, the study of their electronic structure [30], and their first realization in an arc discharge [31], a multitude of methods have been developed for BNNT synthesis (including chemical vapor deposition (CVD) [32, 33, 34], ball milling [35], and laser ablation [36]) and purification which have been reviewed in depth in the literature [37, 38, 39, 40, 41, 42, 43, 44]. To date, thermal plasma has shown to be one of the most promising production methods for its high enthalpy and chemical reactivity as it can, in specific conditions, overcome kinetic barriers needed for BNNT synthesis. This one‐step production method has shown to yield scalable, substrate‐ and catalyst‐free, highly crystalline, and relatively pure BNNTs [45, 46]. This method is based on continuous vaporization of a B‐containing feedstock in a nitrogen‐rich environment to produce BNNTs via nitridation reactions (e.g., 2B + N_2_ → 2BN).

The literature reports on BNNT synthesis in thermal plasma, where the main focus has been on providing kinetically favorable pathways for the notoriously slow B‐N_2_ reaction, employing different approaches such as using high pressure [45], catalyzing the reaction with hydrogen (H) [46], utilizing various B‐containing feedstocks [45, 47, 48], and employing other forms of thermal plasma [49, 50, 51]. The findings indicate that high pressures increase the plasma energy density, residence time, and collision rates of gaseous B‐ and N‐based precursors to successfully form BNNTs [45]. The introduction of H to the reaction environment generates NH and BH species which provide lower‐barrier pathways for BNNT formation compared to relatively inert N_2_ [52]. Replacing hexagonal boron nitride (*h*BN) with ammonia borane (AB) as a feedstock has shown to provide additional H that further improves BNNT formation at atmospheric pressure [47].

The synthesis of BNNTs in thermal plasmas has been discussed to rely on the availability of effective B droplets (i.e., the subset of B droplets that successfully nucleate BNNTs; subsequently referred to as B seeds) as nanotube nucleation sites via the root‐growth mechanism, as it has widely been considered to resemble carbon nanotubes (CNTs) growth [45, 47, 48, 49, 52, 53, 54]. Ex situ transmission electron microscopy (TEM) has routinely been used to show B seeds anchored to one end of a nanotube as evidence for their role in BNNT nucleation. In our previous reports of BNNT synthesis, we observed these seeds to be roughly 10–20 nm in diameter irrespective of the feedstock (*h*BN or AB) under similar conditions [37, 47]. However, the question of controlling B seed morphologies and abundances and their effect on BNNT selectivity and characteristics remained elusive in the context of thermal plasma processes.

Here we processed AB in a nitrogen‐hydrogen‐rich environment in a radio frequency inductively‐coupled plasma (RF‐ICP) system to understand the effect of B seeds on BNNT selectivity. To generate a range of B droplet sizes and quantities, the growth of B droplets was controlled both thermodynamically and kinetically by adjusting B partial pressure and thermal flow fields in the reaction stream. For instance, the feedstock feed rate affects the B partial pressure and by extension its nucleation into B droplets of different sizes and quantities. Varying the plasma power influences the stream fluid dynamics causing variabilities in species mass transport, B concentration, and its reaction rate with N/H radicals, ultimately promoting or suppressing the efficiency of BNNT growth.

Following the synthesis of various samples at different feed rates and plasma powers, the overall purity of these samples was qualitatively assessed using scanning electron microscopy and Fourier transform infrared spectroscopy. Further, thermogravimetric analysis was carried out to estimate their residual B content. TEM imaging was used to analyze the samples and to carry out a rigorous statistical analysis for nanotube diameter, number of walls, and diameters of B particles and B seeds. Through the presented analysis, we show a causal relationship between B seed abundance and BNNT selectivity. Finally, we discuss the morphology of the seed‐nanotube region based on ex situ TEM observation, and propose a growth mode informed by these observations.

## 2 Experimental and Analytical Methods

**FIGURE 1:**
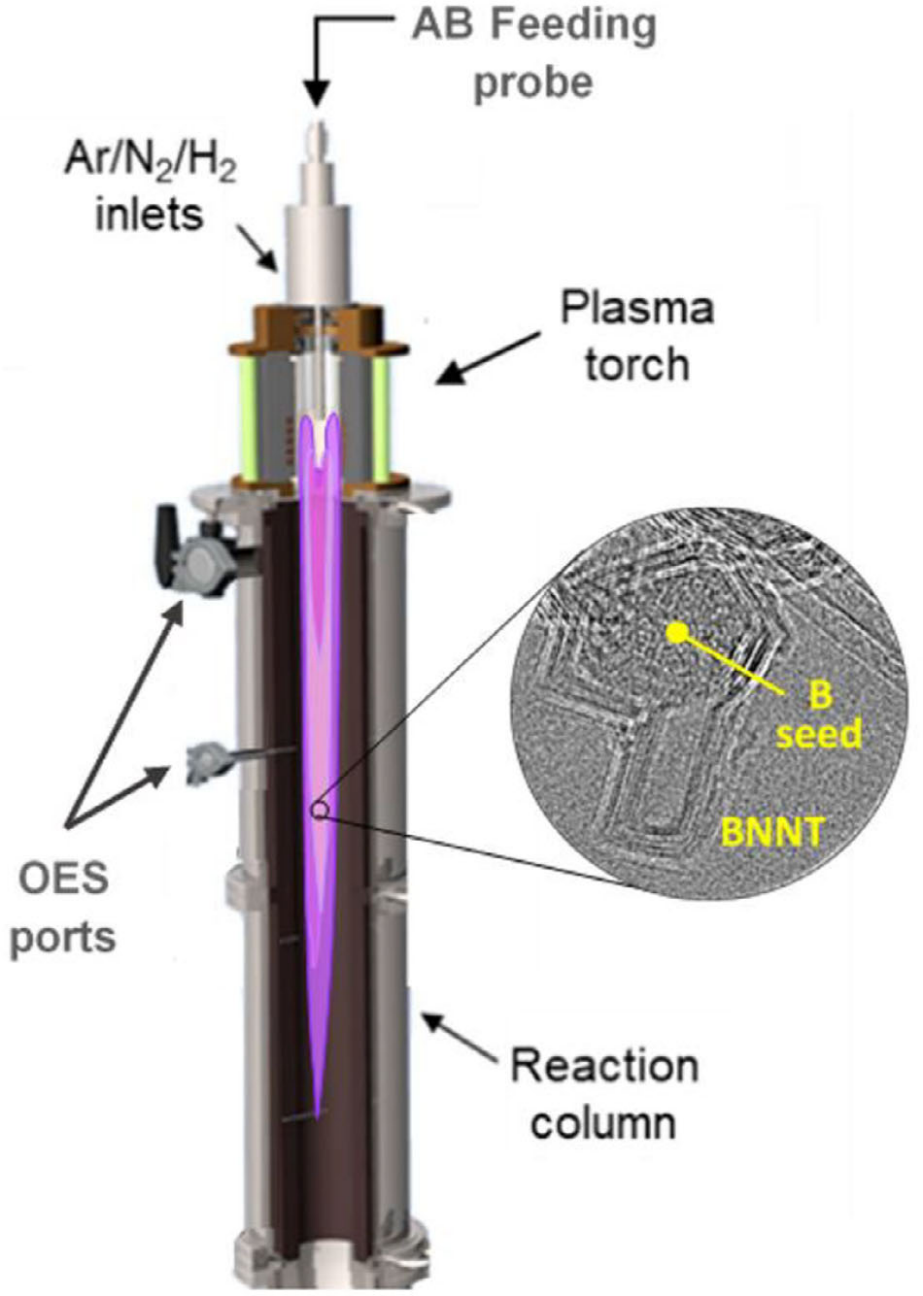
Schematic diagram of the inductively‐coupled plasma system.

**BNNT Synthesis**: The samples were prepared using the hydrogen‐assisted BNNT synthesis (HABS) method [47] using the inductively coupled plasma system shown in Figure 1. The plasma‐forming gas (Ar, 30 slpm) and sheath gas mixture (Ar/N_2_/H_2_: 25/135/60 slpm) are introduced through the torch inlets. The feedstock (ammonia borane, AB, 97%, ∼200 µm, United Boron) is delivered to the plasma flame through a separate axial inlet using a powder feeder (PFV‐100, TEKNA, Canada) and a carrier gas (Ar, 3 slpm). For the controlled growth of B droplets, the feedstock feed rate and plasma input power were varied independently as summarized in Table 1. In the AB feed rate‐dependent experiments, AB feed rate was 0.5, 1, 2, or 4 g/min processed at a plasma power of 38 kW. In the plasma power‐dependent experiments, 28, 38, or 48 kW was used to process AB at 1 g/min. In all the runs, the pressure was kept at 93 kPa. Optical emission spectra (OES) were recorded in the 200–1000 nm range using Ocean Optics spectrometer (Flame S‐XR1‐ES). Spectra were recorded at 7 and 20 cm below the torch nozzle with a 6 and 40 ms integration time, respectively, averaged over 10 scans. The plasma is assumed to be optically thin. Emission intensities of species normalized with respect to intense H_α_ line [47] were compared across different experimental conditions. However, direct species‐to‐species comparisons were not made due to their distinct emissive properties.

**TABLE 1:** Process conditions for BNNT synthesis in the HABS process.

Varied parameter	Fixed parameter	Fixed conditions
AB feed rate: 0.5, 1, 2, 4 g/min	Plasma power: 38 kW	Plasma‐forming gas (Ar): 30 slpm Sheath gas (Ar/N_2_/H_2_): 25/135/60 slpm Carrier gas (Ar): 3 slpm Pressure: 93 kPa
Plasma power: 28, 38, 48 kW	AB feed rate: 1 g/min	

**BNNT characterization**: Field emission scanning electron microscopy (FE‐SEM, Hitachi S4800) and TEM (Thermo Scientific Talos F200X G2) were used to evaluate the morphology and structural characteristics of BNNTs and by‐product impurities. The sample preparation for SEM imaging involved placing a representative sample material directly on a carbon tape adhered on an SEM stub. For TEM imaging, ∼10 mg of the sample was ultrasonically dispersed in deionized water, followed by drop‐casting onto a lacey carbon‐coated TEM grid.

Fourier transform infrared spectroscopy (FTIR, Cary 630, Agilent) was performed in transmission mode to evaluate the overall purity of samples. Thermogravimetric analysis (TGA, Netzsch STG 449 F1 coupled with Bruker Tensor 27 FTIR spectrometer) was performed to estimate the residual boron content and analyze the off‐gases. About 10 mg of each material was heated from room temperature to 1000°C with a ramp rate of 10°C/min in dry‐air conditions to achieve near‐complete boron oxidation.

**Statistical analysis**: TEM images were used to analyze distributions of nanotube diameter, number of walls, and diameters of B particles to compare their variabilities across the sample materials. The diameters of B seeds—a B particle anchored to an end of a nanotube—were measured separately after confirming their attachment to nanotubes. For an adequate sample size, 80–100 TEM images were acquired for each sample using various magnifications to minimize magnification biases. Variations in BNNT selectivity were present across the samples, thus, for ensuring a consistent statistical analysis, equally sized subsets for each distribution were randomly selected and analyzed.

**B nucleation temperature and critical cluster size**: To investigate the effect of B partial pressure on B particle size, the nucleation temperature (T, K) was first calculated using the homogeneous nucleation theory [55, 56] and thermodynamic equilibrium data in Figure S1. Properties of liquid B were taken from the work of Millot et al. [57]. The temperature was then used to calculate the critical size of B clusters (d_pcr_, nm) [58]. The flux of B monomers onto the cluster surface (φ, monomer m**^−^** ^2^ s^−1^) was determined using the Gibbs‐Kalvin relation [59]. (Details are provided in Supporting Information).

**BN formation temperature**: Thermodynamic equilibrium charts in Figure S1 were used to estimate the formation temperature of solid BN at various feed rates. This approach was used instead of the homogeneous nucleation theory due to the lack of surface energy data for BN. The equilibrium calculations were performed using FactSage 8.2 software.

**Residence time, turbulence, and cooling rate**: Computational fluid dynamics (CFD) profiles were approximated from existing data reported in the work of Kim et al. [60], which studied the same system geometry and similar conditions across a range of plasma powers. They were used to understand the flow dynamics in the B droplet growth zone. The axial profiles of temperature and velocity (Figure S2a,b) were used to evaluate the total residence time (${\bar{t}}_{t}$, s) of plasma gas in the system (Figure S2c), the average local residence time (${\bar{t}}_{l}$, s), and the cooling rate (ΔT/Δt, K s^−1^). The turbulent to laminar viscosity ratio profiles (Figure S2d) were used to elucidate plasma power‐induced turbulence on mass transport of B vapor and precursors. (Details are in Supporting Information).

## 3 Results

The selectivity toward BNNTs in the plasma process is closely tied to the size and quantity of B droplets as well as the concentration of BNNT precursors. Following the injection of AB in plasma, it decomposes at relatively low temperatures (above 1500°C) [61, 62]. The plasma extreme temperatures (> 8 000 K) generate a complex mixture of atoms, radicals, and meta‐stable B_x_N_y_H_z_ intermediate species from the feedstock and the sheath gas flowing at the periphery of the plasma jet. Convective, diffusive, and thermophoretic processes drive these species to cooler regions. Then, B vapor reaches supersaturation, leading to homogeneous nucleation forming sub‐nano nuclei of a critical size. Stable nuclei grow into B droplets and, under a window of residence time, turbulence, and cooling rates, can support nanotube nucleation. Effective B droplets that support BNNT growth, typically found anchored to an end of nanotubes, are hereafter referred to as B seeds. Ineffective B droplets, which fail to sustain nanotube nucleation, condense as B particles. The abundance of B seeds and BNNT precursors is necessary for nanotube nucleation, and their imbalance leads to competing pathways for forming non‐tubular BN morphologies.

### 3.1 Effect of AB Feed Rate on BNNT Selectivity

**FIGURE 2:**
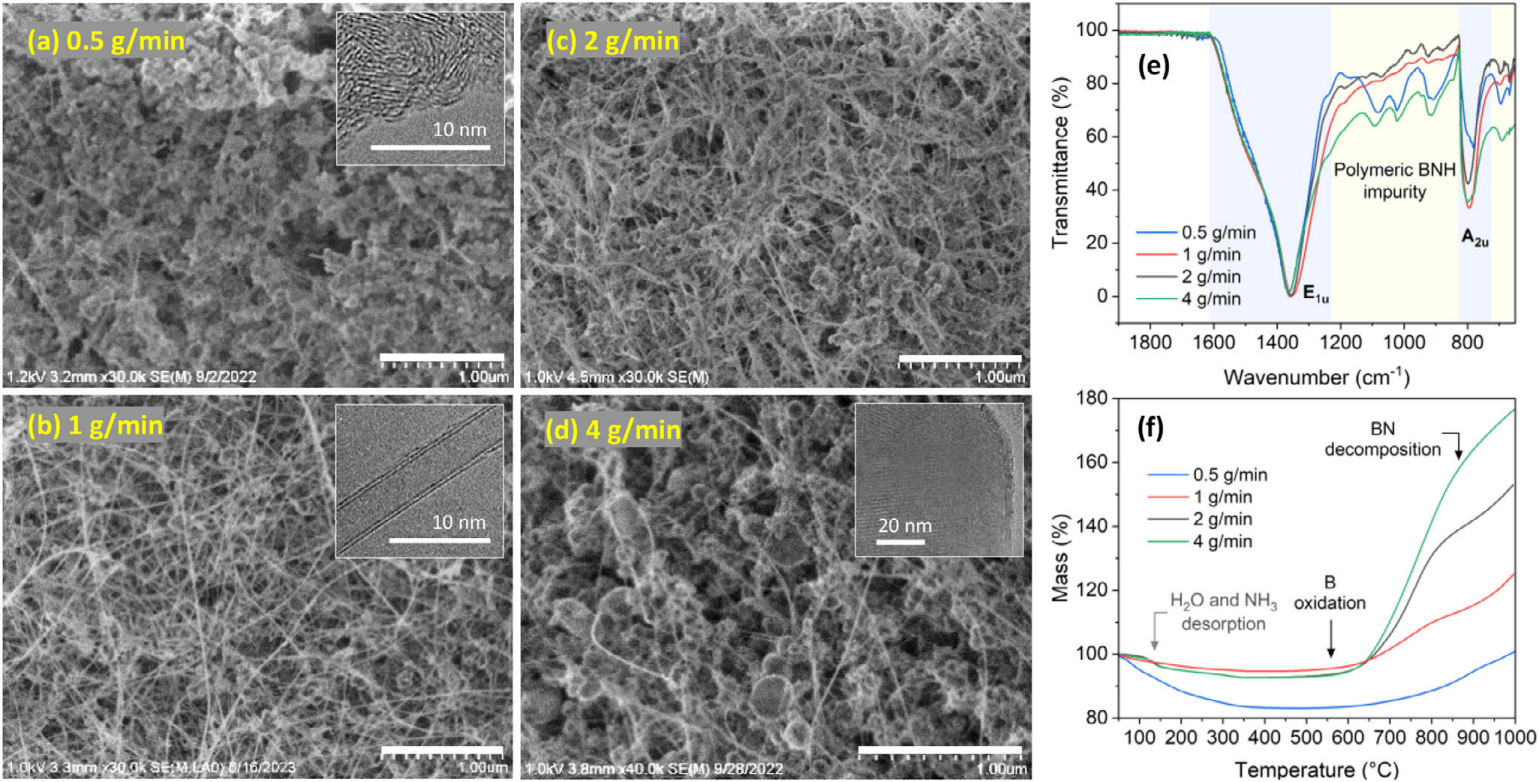
(a–d) SEM images for BNNTs made at 0.5, 1, 2, and 4 g/min AB, respectively (scale bar: 1 µm), and their associated (e) FTIR and (f) TGA spectra. Insets in (a), (b), and (d) respectively show TEM images of a non‐tubular BN particle, double‐wall BNNT, and BN‐coated B particle.

AB feed rate significantly affected BNNT selectivity, which was reflected in the macro texture and color of the as‐produced materials. To qualitatively assess the BN morphologies, we primarily rely on SEM/TEM observations as the interest is to highlight the overall prevalence of tubular versus non‐tubular structures. SEM images in Figure 2a–d show an initial increase in BNNT presence, followed by a decrease as feed rate was further increased. At 0.5 g/min, the product is mostly composed of flake‐like *h*BN, turbostratic BN (*t*BN), and amorphous BN (*a*BN) particles, BN‐encapsulated B particles, and a negligible presence of BNNTs. At 1 g/min AB, a major shift in morphology is observed in favor of BNNTs, seen as dense web‐like nanotube networks with a minimal presence of other particles. B particles encapsulated with graphitized BN layers increase in quantity and size as AB was increased to 2 g/min, becoming nearly prevalent at 4 g/min. Therefore, microscopy suggests that the optimal feed rate is 1–2 g/min. A detailed description of these B and BN morphologies can be found in our previous study [47].

From the low‐frequency FTIR spectra shown in Figure 2e, the peak position corresponding to the in‐plane vibrational mode, E_1u_, is the lowest for the 1 and 2 g/min samples (1352 cm^−1^), correlating with their high purity [63] due to reduced *t*BN and *a*BN impurities. Narrow E_1u_ peaks have been attributed in the literature to reduced *h*BN impurity [64]. However, peak analysis of at least 5 scans (not shown) revealed that, in general, the width of this peak was relatively constant across the samples, providing limited basis for conclusive assertions. Samples prepared at 1 and 2 g/min also showed intense peaks for the out‐of‐plane vibrational mode, A_2u_ (795 cm^−1^). This may reflect the sample's degree of crystallinity, relating to a decreased presence of BN impurity (e.g., *t*BN/*a*BN). The two samples also showed a decrease in intensity of peaks attributable to polymeric BNH impurities as indicated by the multiple peaks between E_1u_ and A_2u_.

The residual B content was estimated using TGA as shown in Figure 2f. The corresponding derivative thermogravimetric (DTG) curves and FTIR spectra for evolved gaseous byproducts at specific temperatures are shown in Figure S3 along with a relevant discussion. Pure B is known to begin to oxidize below 400°C when in direct contact with an oxygen‐rich atmosphere [64]; however, the encapsulating BN shells delayed the oxidation to about 600°C as observed in the thermogram. Then, it increased rapidly above 700°C and decelerated at 800°C. Defective/low‐crystalline BN starts to decompose at ∼850°C [65] generating oxidizable B. The residual B mass percentage, which was calculated based on the assumption of complete oxidation of B particles into B_2_O_3_, increased nearly linearly with AB feed rate (8, 14, 27, and 38 wt.%, respectively). These values will be used to identify the sample with the highest B seed abundance relative to other B particles.

**FIGURE 3:**
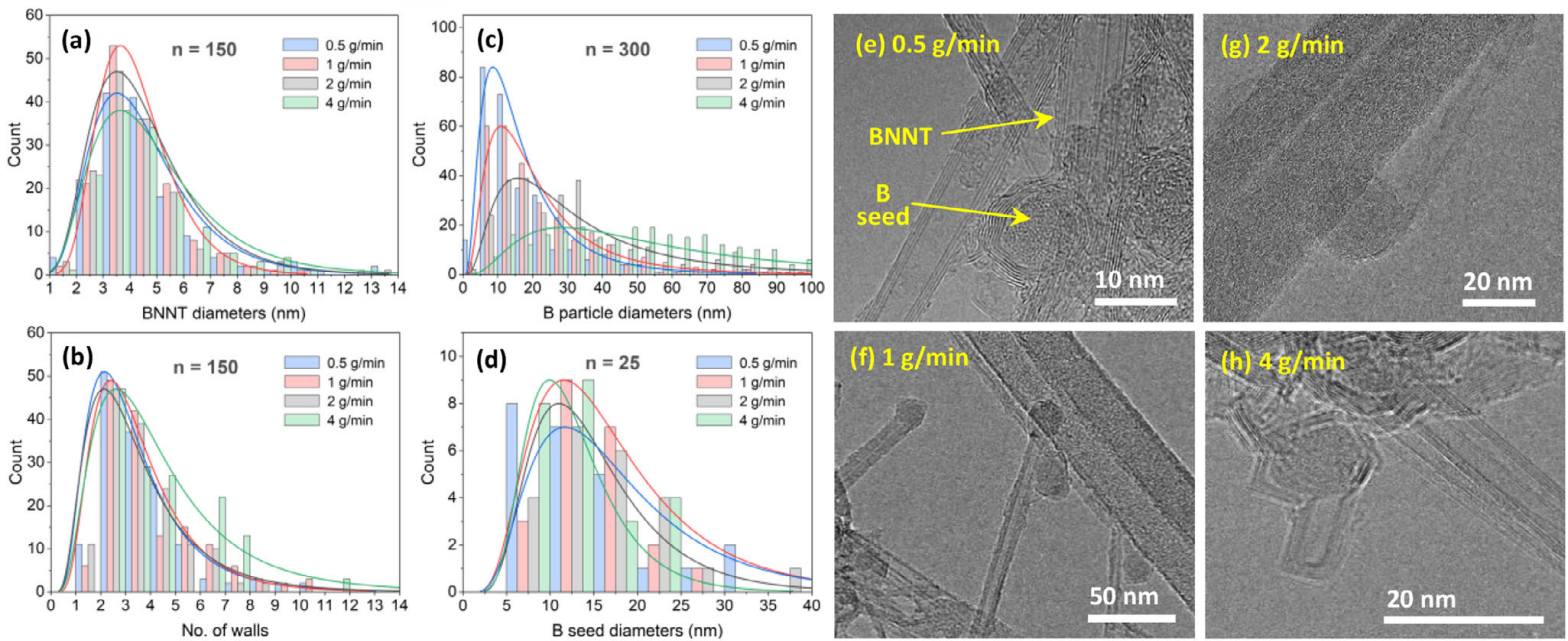
The effect of AB feed rate on distributions of (a) BNNTs diameter, (b) number of walls, and the sizes of (c) B particles, and (d) B seeds. The histograms represent the counts and the curves are their corresponding lognormal fits. (e–h) Typical TEM images for B seeds anchored at an end of a nanotube formed at 0.5, 1, 2, and 4 g/min AB, respectively.

A survey of TEM images revealed that the nanotube diameters and number of walls remained largely unaffected by varying the feed rate (3‐5 nm and 2–4 walls, Figure 3a,b, respectively), while the average size of B particles gradually increased (Figure 3c). The change in B particle size and the associated decline in BNNT selectivity while maintaining nanotube characteristics indicates that B seeds are indeed critical for BNNT nucleation. Thus, controlling their size and abundance can optimize BNNT selectivity. In that context, quantifying B seeds is important. To do so, ex situ TEM imaging was first used to identify the size range of these seeds, which was found to be approximately 7–20 nm for the four feed rates (Figure 3d); typical examples are shown in Figure 3e–h. This range, determined using the Highest Density Interval method, encompasses ∼70% of the seed population, and therefore was used as the boundary of integration (x_min_ and x_max_) for the area under the curve of B particle distributions in Figure 3c (these distribution curves are defined by f(x) in Equation S12). This area represents the count of B seeds relative to other B particles. The resulting value was then multiplied by the corresponding residual B ratio (R_B_) found from the TGA analysis. This estimates B seed abundance (N_seed_), as described by Equation 1. As listed in Table 2, the 1 and 2 g/min samples showed the highest B seed abundance by a significant margin compared to 0.5 and 4 g/min. This weighted metric reflects, as a first‐degree approximation, the contribution of B seeds relative to other B particles, though it should be interpreted only as a pattern rather than definitive values. Notably, this pattern is consistent with the SEM observations and FTIR purity indication data, suggesting a relation between the abundance of B seeds and BNNT selectivity.

(1) $$N_{\mathrm{seed}}=R_{B}\underset{x_{\min}}{\int^{x_{\max}}}f\left(x\right)\mathrm{dx}$$

**TABLE 2:** Residual B ratio, B seed size range, and estimated relative B seed abundance for BNNT samples obtained at various AB feed rates.

AB feed rate (g/min)	Residual B ratio, R_B_	B seed size (nm), Integration boundaries (x_min_ ‐ x_max_)	Relative B seed abundance (count), N_seed_
0.5	0.08	7‐20	55
1	0.14	7‐20	100
2	0.27	7‐20	120
4	0.38	7‐20	60

Because the B particles are encapsulated with BN shells, it is possible that the TGA analysis presented above could have underestimated the residual B content. In addition, the high temperature may have caused oxidation of amorphous and poorly crystalline BN particles, potentially overestimating the residual B content. To address these issues, we performed another TGA analysis in which the samples were heated from room temperature to 700°C and held for 20 h. Similar mild temperatures and extended oxidation periods have routinely been shown to effectively oxidize BN‐encapsulated B particles [66]. The relative B seed count derived from this TGA analysis showed a trend consistent with the results presented above. The results are shown in Figure S4 and Table S2.

**FIGURE 4:**
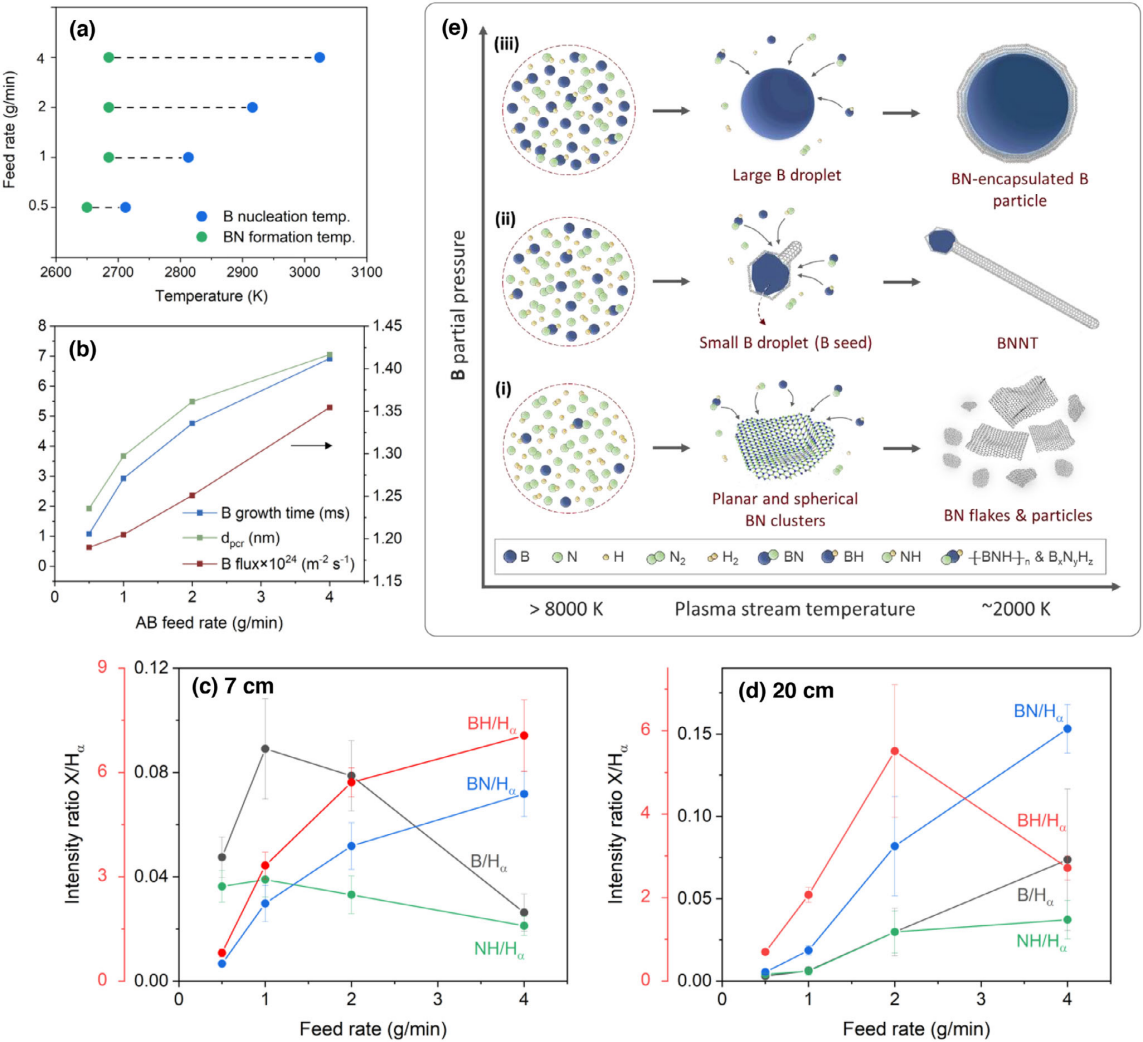
The effect of B partial pressure, related to AB feed rate, on (a) B nucleation temperature shown relative to BN formation temperature, and (b) B cluster critical size, B monomer flux, and B droplet growth time. OES intensities of B, BN, NH, and BH relative to H_α_ for various feed rates acquired at (c) 7 and (d) 20 cm below the torch nozzle. Red y‐axis corresponds to BH/H_α_. (e) Schematic diagram illustrating BNNT selectivity dependence on B partial pressure in a plasma stream.

B seed abundance is directly affected by the feed rate, which influences the final size and quantity of B particles. To understand this influence, we studied the growth time, critical diameter of B nucleus, and B monomer flux during the B droplet formation for each feed rate. To estimate the B droplet growth time, we first estimated the B nucleation using the nucleation theory, and the BN formation temperatures from thermodynamic equilibrium charts in Figure S1. These temperatures are shown in Figure 4a. It is noteworthy that the nucleation temperature of B increases with the feed rate while the BN formation temperature is nearly unchanged. With the assumption that B droplets growth commences at the B nucleation temperature and ceases at the BN formation temperature (where BNNT starts to form), this range was used to estimate the B growth time from the calculated temperature and velocity profiles in Figure S2a,b for the 38‐kW case. The resulting B droplet growth time is shown in Figure 4b, which increases with the feed rate.

The nucleation temperatures were also used to estimate the critical diameter of the B nucleus as well as the B monomer flux (Figure 4b). Similar to the trend observed for the B droplet growth time, the critical diameter and the B monomer fluxes increase with the feed rate. These three factors contribute with a direct proportionality to the final size of B droplets, thus explaining the size distributions in Figure 3c. While a high B partial pressure increases the B droplet count, an ‘excessively’ high partial pressure leads to overgrowth of droplets caused by longer growth time and higher monomer flux, making them unsuitable as B seeds for BNNT growth.

The emission intensity of B (I, 249.9 nm), NH (A ^3^Π−X ^3^∑^−^, 336.2 nm), BN (A ^3^Π−X ^3^Π, 360.1 nm), BH (A ^1^Π−X ^1^∑, 433.2 nm) relative to H (α, 656.6 nm) [67, 68] were tracked through OES at 7 and 20 cm below the torch nozzle, which are close to the AB dissociation and B seed/BNNT nucleation zones, respectively. The existence of these intermediates in this thermal plasma process was discussed in detail in our previous work [46], and was further validated in the following works [47, 52, 60]. Representative spectra are shown in Figure S5. The objective of analyzing OES spectra is to observe the trend of B emission to qualitatively support the discussion above on B monomer flux, as well as to observe the trend of emission of other species as they are essential for BNNT formation. The species relative emission intensities are summarized in Figure 4c,d. Near the torch, the highest emission for B was observed at 1–2 g/min. At these feed rates, the emissions from BN and BH are also high. At 4 g/min, AB dissociation into its elements was low, evidenced by the high emission of BN and BH and the low emission of B. This is consistent with the BNNT selectivity being affected by the available free B along with the other BN, BH, and NH precursors. Downstream, the emission of B is seen to increase with the feed rate due to further dissociation, and that is consistent with the B monomer flux, which also increased with the feed rate. B emission is the highest at 4 g/min, however, the emission of other species is generally not as high. At 1–2 g/min, on the other hand, the emission of B as well as the other precursors are all the highest. These findings suggest that 1–2 g/min facilitate, broadly speaking, the highest combination of B_x_N_y_H_z_ species while also maintaining a high presence of free atomic B.

Putting together the findings of TEM‐based statistics, B partial pressure calculations, and OES observations, one can reasonably arrive at the following interpretations. At 0.5 g/min, B droplets are the smallest in diameter and have the narrowest distribution and lowest number density, due to the partial pressure of B being the lowest. This condition results in shifting the B droplet diameters to be smaller than the 7–20 nm range, therefore decreasing BNNT selectivity. The selectivity also decreased in the 4 g/min case due to the higher B partial pressure, shifting B droplets to mostly grow larger than the 7–20 nm range. These large B droplets are suitable for BN‐encapsulation rather than BNNT growth. The partial pressure of B at 1–2 g/min is optimal under which 7–20 nm droplets are formed with the highest number density, while maintaining a high number of B_x_N_y_H_z_ intermediates, thus the BNNT selectivity is also the highest. These interpretations are schematically described in Figure 4e.

### 3.2 Effect of Plasma Power on BNNT Selectivity

**FIGURE 5:**
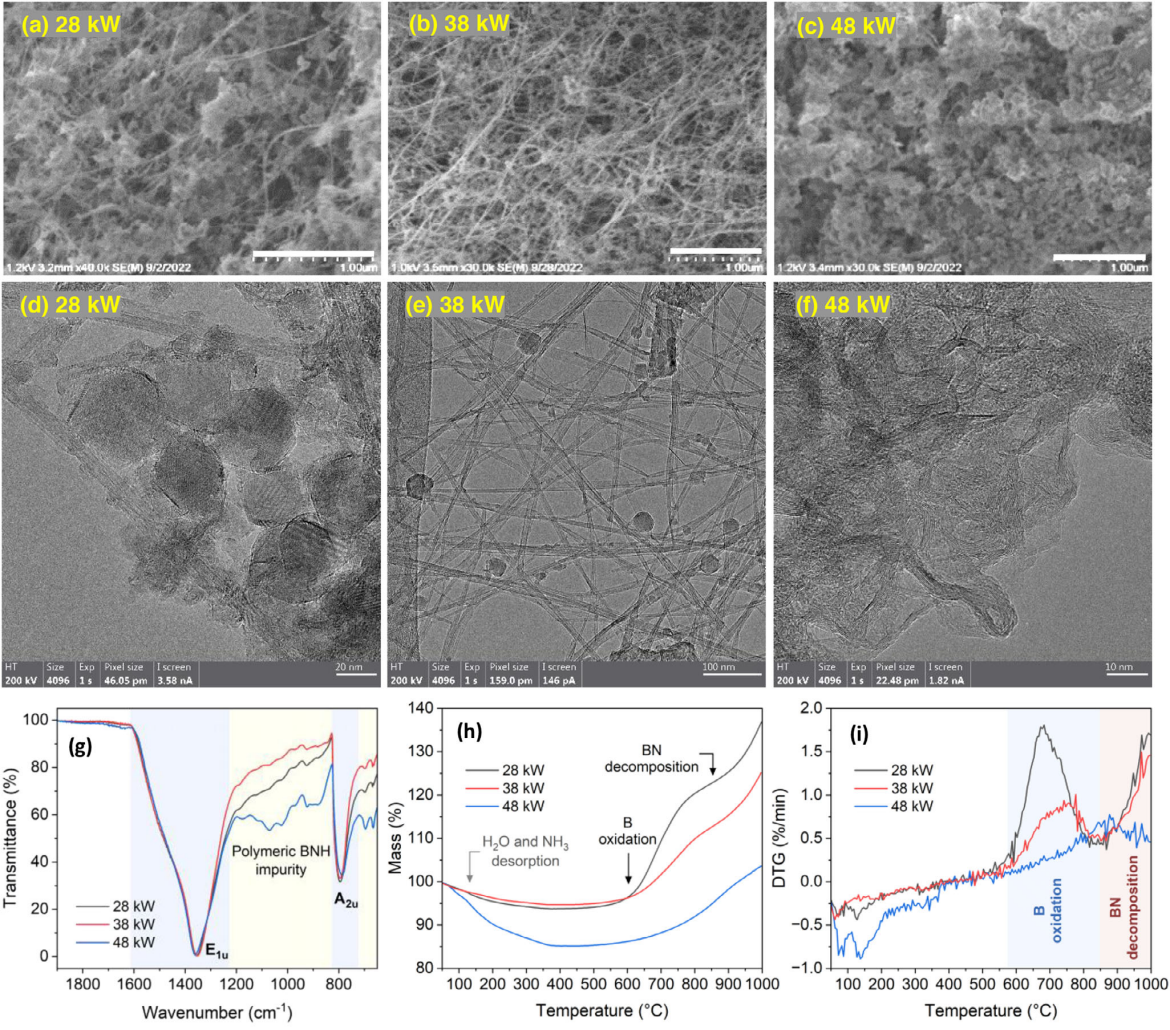
(a‐c) SEM and (d–f) TEM images of BNNT materials synthesized at 28, 38, and 48 kW, respectively. (g) FTIR, (h) TGA, and (i) DTG are spectra corresponding to these materials.

Selectivity toward BNNTs is highly sensitive to power adjustments. Through SEM images (Figure 5a–c), we observed a clear trend of an increase followed by a decrease in BNNTs as power increases, 38 kW being the optimal power. The chemical nature of impurities was similar across the samples, but varied in their quantities. TEM images (Figure 5d–f) provided insights into the material microstructural characteristics, in which careful examinations revealed that polydisperse BN‐encapsulated B particles were more prevalent in the 28‐kW than in the 38‐ and 48‐kW samples. Both crystalline and amorphous B particles were present with no apparent dominance. At 48 kW, we observed that multilayered BN nanosheets and other non‐tubular BN structures were more prevalent compared to the other two powers. This suggests that there was a decreased presence of B seeds, which induced homogeneous nucleation of BN species into non‐tubular BN microstructures. Additionally, the presence of BNH polymeric byproducts was the lowest at 38 kW, as indicated by the FTIR spectrum in Figure 5g. These species are revealed through the peaks between the vibration modes of B‐N stretching (E_1u_) at 1360 ± 4 cm^−1^ and B‐N‐B bending (A_2u_) at 790 ± 2 cm^−1^. TGA results (Figure 5h,i) corroborate observations from microscopy: the residual B mass ratio decreased with increasing plasma power (20, 14, and 8 wt.%, respectively). It is worth noting that the residual B content was the highest at 28 kW, suggesting that this low power is not a bottleneck for vaporizing/decomposing the feedstock. Also, at 48 kW, the decomposition rate of BN structures above 850°C is low, indicating a significant presence of highly crystalline BN nanosheets.

**FIGURE 6:**
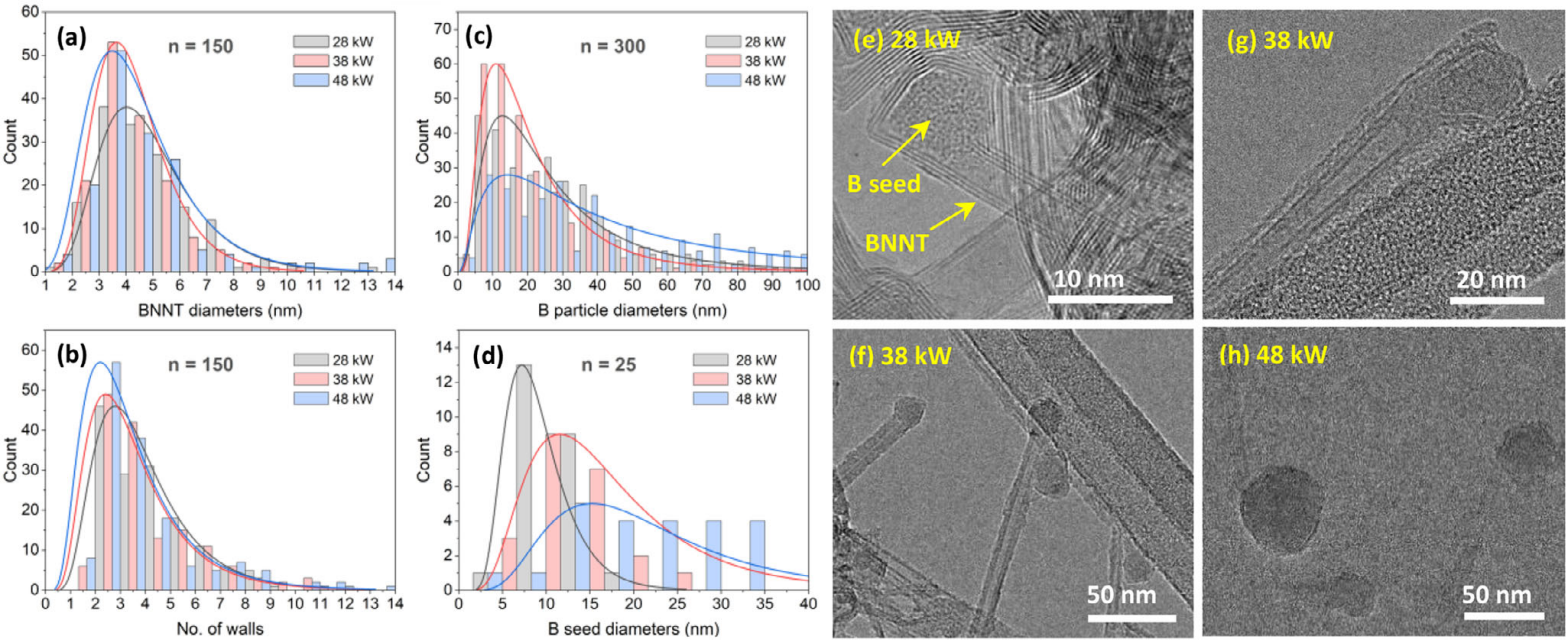
Effect of plasma power on the distributions of (a) BNNTs diameter, (b) number of walls, and sizes of (c) B particles and (d) B seeds. (e–h) Typical TEM images for B seeds anchored at an end of a nanotube formed at 1 g/min and powers of 28, 38 (two images) and 48 kW, respectively.

The produced BNNT materials maintained similar structural characteristics regardless of plasma power. Similar to AB feed rates samples, the majority are 3–5 nm in diameter (Figure 6a), and possess 2–4 walls (Figure 6b). Meanwhile, the diameter of B particles (Figure 6c) changed, as indicated by the lognormal mode, to be the smallest and narrowest for 38 kW followed by 28 and 48 kW (∼10, 12, 15 nm, respectively). Interestingly, the size of B seeds increased with power (Figure 6d); typical TEM images are shown in Figure 6e–h. At each power level, the majority (∼70%) of the seeds are within specific size ranges: 5–10 nm at 28 kW, 7–20 nm at 38 kW, and 10–25 nm at 48 kW. The change in power clearly promoted diverse sizes for B particles and B seeds while the characteristics of the nanotubes remained unaffected. This indicates again that B droplets of a specific size range (B seeds) are critical for BNNT nucleation, and a high abundance is required for a high BNNT selectivity. To estimate the abundance of B seeds, we utilized the same method outlined in Section 3.1, but adjusted the integration boundaries (x_min_ and x_max_) accordingly. The estimation used B particle and B seed size distribution curves (Figure 6c,d, respectively), and the residual B ratio (R_B_) from the TGA results (Figure 5h). The B seeds abundance (N_seed_) was found to be the highest, by a significant margin, in the 38‐kW sample (Table 3). This is consistent with the apparent amount of BNNTs observed in the SEM images and the purity indications from the FTIR data, highlighting once again the existence of a B seeds‐BNNT selectivity relation. A similar pattern was observed with residual B ratios obtained from TGA for the samples heated from room temperature to 700°C and held for 20 h (Figure S4 and Table S2).

**TABLE 3:** Residual B ratio, B seed size range, and estimated relative B seed abundance for BNNT samples obtained at different plasma powers.

Plasma power (kW)	Residual B ratio, R_B_	B seed size (nm), Integration boundaries (x_min_ ‐ x_max_)	Relative B seed abundance (count), N_seed_
28	0.2	5‐10	35
38	0.14	7‐20	100
48	0.08	10‐25	30

**FIGURE 7:**
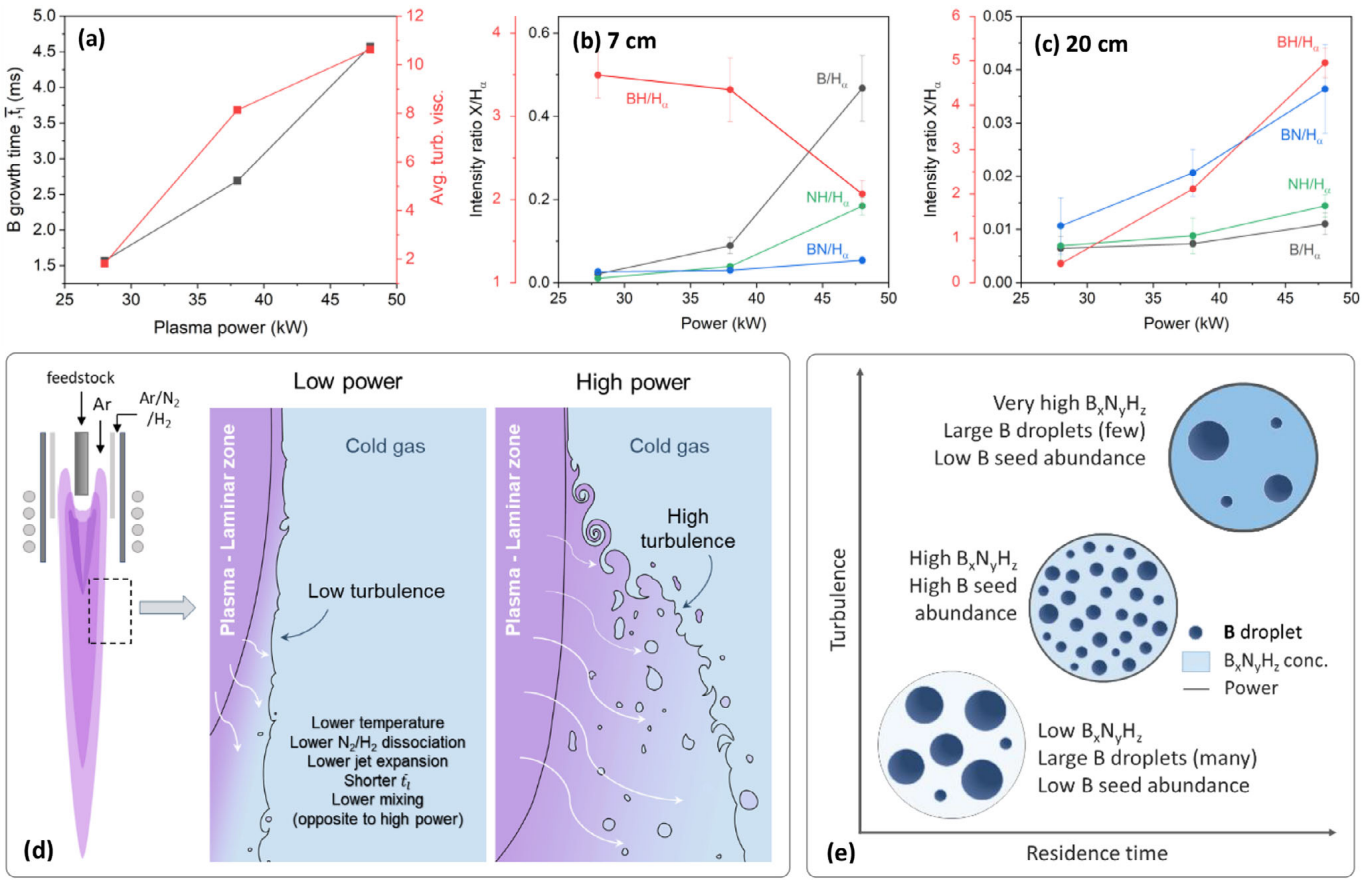
(a) B droplet growth time and average turbulent‐to‐laminar viscosity ratios in the B droplet growth zone along the axis of symmetry. Evolution of relative OES intensities of B, BN, NH, and BH with respect to plasma power acquired at (b) 7 and (c) 20 cm below the torch nozzle. Red y‐axis is related to BH/H_α_. Schematics illustrating (d) the effect of plasma power on turbulence intensity and jet expansion, and (e) the effect of residence time and turbulence on the formation of B droplet/seed as plasma power increases. The background blue shades in the circles reflect B_x_N_y_H_z_ concentration, light for  low and dark for high concentrations; and the thickness of the line enclosing each circle represents plasma power, increasing from low to high.

The abundance of B seeds of a specific size range is influenced by the B particles distribution and the number density. As seen in Figure 6c, the average B particle size is the smallest at 38 kW but larger at 28 and 48 kW. However, the residual B % decreases monotonically with power (Figure 5h), which correlates with a reduction in particles number density. This results from the complex dynamics of B droplet formation prior to nanotube nucleation. In the B droplet growth zone, plasma power influences thermofluid fields, including temperature and velocity. Both factors create variability in the residence time and turbulence intensity in that zone. With increasing power, plasma jet expands providing successively longer B growth times (Figure 7a). This should theoretically result in an increase in the droplet size as condensation of B vapor on droplets surfaces lasts longer. However, the turbulence in this zone, as inferred from the average turbulence to laminar viscosity ratio (Figure 7a), also increases with power [60, 69, 70], and that counteracts the droplet growth. This intensified mixing drives B atoms away from homogeneous condensation on B droplets toward forming B_x_N_y_H_z_ species. This competing effect of prolonged growth time (growth promoter) and intensified turbulence (growth suppressor) is consistent with the high‐low‐high pattern in B particle size distributions observed with increasing plasma power. Likewise, the decreasing mass of residual B as power increases can be explained by those two factors. The low residence time at the low power hinders the kinetically sluggish reaction of B + N → BN to proceed at a high rate, and the low turbulence makes it take place at a lower frequency giving a preference for more B to condense as B droplets rather than as BN. The opposite is expected at the high power where the BN mass becomes high at the expense of B mass.

Another factor that led to those B particle distributions and number density is the extent of dissociation of N_2_ and H_2_ in the sheath gas, which dictates the availability of N and H to react with B. Their high availability (and the level of turbulence) affects reactions such as B + N → BN, N + H → NH, and B + H → BH to proceed to the right. The same OES analysis described earlier was performed at different powers to probe the species evolution under varying dissociation conditions. Representative spectra are shown in Figure S6. The progression of emission intensities of B, NH, BN, and BH relative to H is shown in Figure 7b,c. Near the torch, B emission increases with power consistent with increasing population of free B. Also, the increased emission of NH with power is indicative of enhanced dissociation of N_2_ and H_2_. The rise in free B, H, and N atoms leads to the elevated formation of BH and BN downstream at 48 kW as seen at 20 cm. This indicates an active B‐N‐H chemistry that consumes B at the expense of condensing into B clusters, consequently reducing the B droplet/seed count. This 48‐kW power results in low BNNT selectivity and the appearance of BN nanosheets. Conversely, at 28 kW, emissions of B and BN are weak at both elevations, suggesting that B condenses mostly as droplets. Although abundant, their large diameters and their existence in an environment of low BNH‐based species results in limited BNNT formation.

The schematics in Figure 7d describe the power‐dependent changes in jet expansion (residence time) and turbulence (mixing intensity). Low versus high turbulence near the jet at low and high powers, respectively, is inferred from the calculated turbulence viscosity ratios in Figure 7a. Similarly, the low versus high dissociation degree of N_2_/H_2_ gases under the low and high powers, respectively, are supported by the OES results. Figure 7e summarizes the effect of residence time and turbulence on B droplet formation. As power rises (indicated by the line thickness of the enclosing circles), both parameters increase. Based on OES findings, the B_x_N_y_H_z_ concentration (indicated by the blue shade in the circles) also increases with power. The population and size of B droplets/seeds (dark blue circles) vary accordingly—large and abundant at 28 kW, large but sparse at 48 kW (as confirmed by the TEM and TGA). At a moderate power (38 kW), however, there is an optimal condition where these droplets are sufficiently small and abundant, and coexist with a relatively high B_x_N_y_H_z_ concentration. These conditions, with a moderate residence time and turbulence, favor BNNT selectivity.

## 4 Discussion

The results above help to quantify the importance of B seeds toward nucleation of BNNTs by identifying a clear range of seed particle sizes over which BNNTs appear to be the favored product, and by demonstrating the abundance of such seeds can be manipulated to optimize BNNT yield. Overall, the nucleation of BNNTs shows selectiveness toward B seeds of a size range within ∼5‐50 nm. It is close to the ∼5‐30 nm range of metal catalysts diameters reported in CNTs synthesis in thermal plasmas [71, 72, 73]. In BNNTs, below or above the 5–50 nm range, nanotube nucleation does not seem to be sustainable. Below 5 nm, the B droplet is probably too small to capture B_x_N_y_H_z_ precursors, and it solidifies rapidly. Above 50 nm, the droplet is energetically very stable and lacks edge‐states on its surface to facilitate nanotube nucleation, thus, the precursors favor forming semi‐flat BN facets. Further, within the 5–50 nm range and depending on process conditions, nanotube nucleation shows selectiveness toward narrower B seed size ranges driven by the thermodynamics and kinetics of the process. For example, increasing the B partial pressure increased B particles size, yet the B seeds maintained nearly consistent size (7–20 nm). In contrast, increasing the turbulence intensity and the residence time in the B droplet growth zone resulted in nearly consistent B particles size, but B seeds sizes increased gradually (5‐10, 7–20, and 10–25 nm).

**FIGURE 8:**
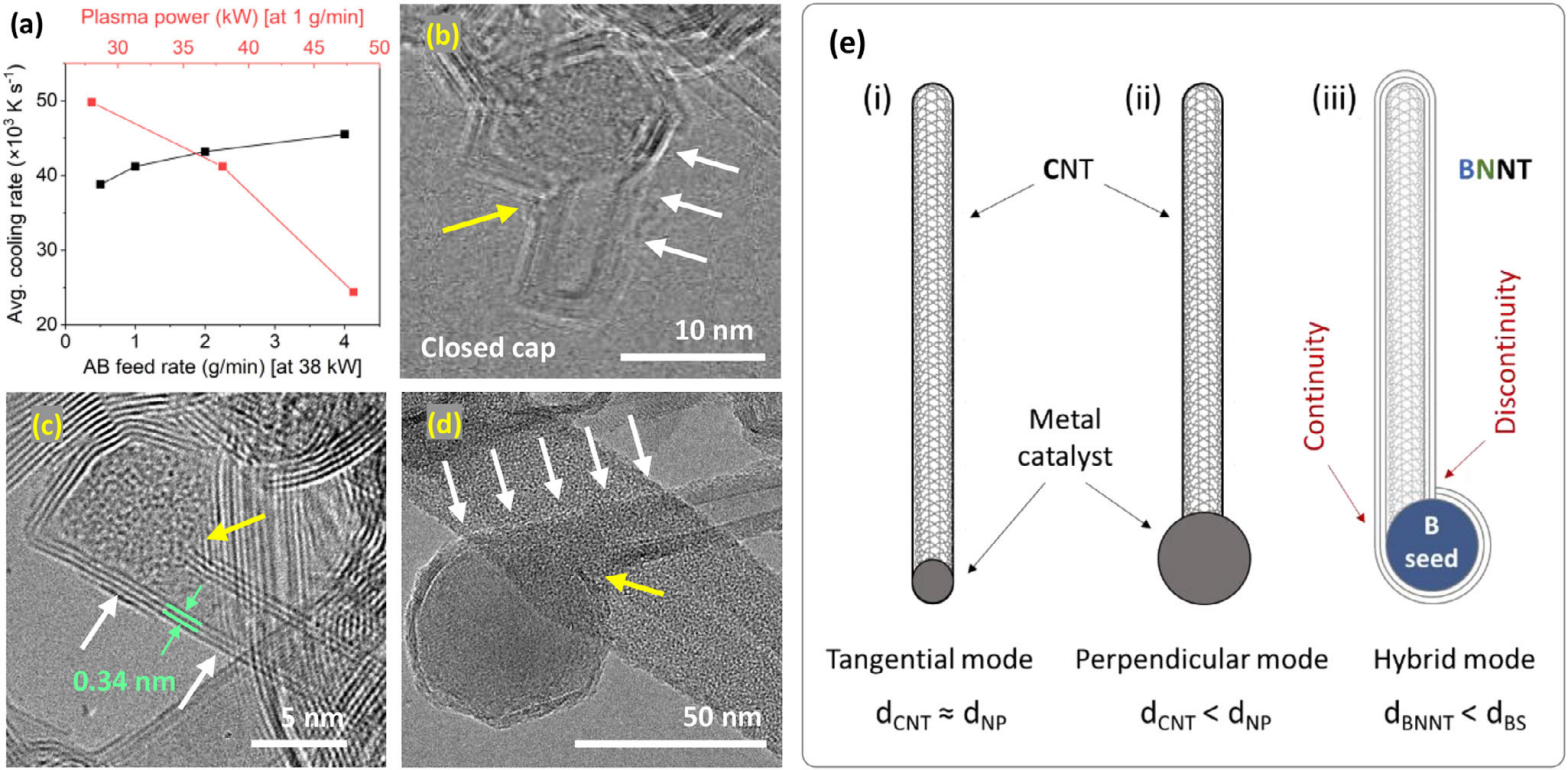
(a) Average cooling rates in B droplet and BNNT formation zones with respect to AB feed rate (black) and plasma power (red). (b–d) BNNT‐seed golf club structure of different seed diameters. White arrows in (b) and (d) show layer continuity between the BN shell and the nanotube, while the yellow ones show the discontinuity. (e) Schematic diagram of tangential, perpendicular, and hybrid modes of nanotube growth. It also indicates the shell‐tube layer continuity in the tangential side and discontinuity in the perpendicular side.

The comparison between the size distributions of B seeds to B particles suggests that the seeds are not merely smaller subsets mirroring the B particle distributions. Rather, nanotube nucleation shows seed‐size sensitivity depending on thermal and kinetic conditions. Under optimal conditions, B seed distribution aligns more closely with B particles distribution, increasing the seed abundance and thereby increasing the BNNT selectivity. This alignment can likely be correlated with the cooling rate at the moment a BNNT nucleates, approximated by the average cooling rate in the B droplet and BNNT formation zones. Cooling rates govern the B droplet solidification rate and B_x_N_y_H_z_ precursor diffusion and interaction on its surface. In Figure 8a, the cooling rate increased slightly with the feed rate, while the B seeds size reduced also slightly (Figure 3d). This effect is more obvious with the power increase; the cooling rate decreased substantially leading to an increase in the B seed size (Figure 6d). This increase (or the decrease in the case of low power), despite the relatively unchanged B particle distributions, suggests that local thermal gradients control the activation stage of B droplets at the moment B_x_N_y_H_z_ precursors are available. So, at high cooling rates (i.e., at low power) smaller droplets become active earlier, and thus dominate the nucleation environment. While at high powers, the low cooling rate shifts the activation to a later time by which point the B droplets have gained more volume, thus becoming the dominant seeds in the nucleation environment. This ability to control the seed size provides an opportunity for improving BNNT selectivity at the non‐optimal conditions by increasing the B seeds abundance in the desired size range. This can be achieved by a simultaneous control of feed rate and power. For example, processing AB at 0.5 g/min at a low power (or 4 g/min at high power) is expected to shift the B seeds toward smaller (or larger) diameters, aligning with the corresponding smaller (or larger) B droplets size. This strategy was tested and confirmed experimentally, and the resulting materials showed a notable improvement in BNNT selectivity as evidenced by the SEM images in Figure S5a,b.

Variability in B seed diameter did not significantly affect BNNT diameters or number of walls. Similar BNNT characteristics have also been reported under different synthesis conditions including pressure [45], reactor geometry [45], feedstock material [45, 46], and ratios of reactive gases [48]. The diameter consistency may be explained by the growth mode. TEM images (e.g., Figure 8b–d) reveal a high prevalence of nanotubes extending from B seeds in a *golf club‐like* configuration irrespective of seed diameter. A fully formed BNNT showing its two ends (seed and closed cap) is presented in Figure S8. This morphology has also been discerned in TEM/STEM images from previous BNNT synthesis in CVD processes [74, 75, 76, 77], although they were only shown incidentally and not structurally analyzed in detail. It is worth noting that the BNNT diameters reported in CVD studies differ from the ones reported in this work. Such variations are expected given the substantial differences in the thermodynamics and reaction kinetics between the two synthesis methods.

The golf club‐like structure features a multilayered polyhedral BN shell encapsulating the seed, with a nanotube extending laterally. This growth mode is a hybrid of tangential and perpendicular modes, commonly discussed in the context of carbon nanotube (CNT) growth on metal nanoparticles [78, 79, 80] and observed in BNNT grown in CVD and metal‐catalyzed plasma‐assisted CVD [81, 82]. In the tangential mode, the CNT wall is oriented tangentially to the nanoparticle surface, resulting in d_CNT_ ≈ d_NP_, while in the perpendicular mode, the wall is aligned perpendicularly, resulting in d_CNT_ < d_NP_ or no clear relationship. In the present BNNT case, we observe a *hybrid mode*, in which one side is aligned tangentially to the seed surface and gradually transitions to be perpendicular on the other side (Figure 8c). The perpendicular component is likely responsible for minimizing the dependence of nanotube diameter on the seed diameter, leading to d_BNNT_ < d_BS_. Schematics of the modes are presented in Figure 8e.

Furthermore, Kim et al. [83] proposed two models for BNNT growth in a high‐pressure laser system. In the first model, nitrogen molecules penetrate the B seed surface to form a BN cap that subsequently grows into a closed‐end nanotube via the root‐growth mechanism, followed by BN encapsulation. In the second model, a partial BN shell first forms around the seed, and then precursors react with the shell's open part extending it into an open‐end nanotube. Thus, the layers of the nanotube wall are connected continuously with the layers of the shell. In our system, however, we observed a closed‐end nanotube with dual connections at its base: continuous with the BN layers at the tangential side and discontinuous at the perpendicular side (Figure 8b–d,e(iii)). We also observed that the nanotube number of walls does not always equal the BN shell layers, except at the layer continuity region. For instance, they closely match in Figure 8b,c, but differ in Figure 8d. Notably, they tend to converge as the seed size reduces.

The assembly of the golf club‐like structure featuring the hybrid growth mode, the shell‐tube dual connectivity, and the shell‐tube thickness mismatch can plausibly be explained by the following formation pathway, which is inferred from post‐synthesis TEM observations. Upon seed growth maturity, B_x_N_y_H_z_ precursors interact with its surface, forming a BN island. A local asymmetric protrusion develops, characterized by being off‐centered and tilted relative to the seed surface, forming the base of the golf club‐like configuration (white arrows in Figure 8b,d). The protrusion develops as a result of competing forces that aid the liftoff, namely, structural stresses caused by non‐hexagonal defects and adhesive forces between the BN phase and the seed surface. The asymmetric geometry is likely caused by a local sharp curvature on the seed, and assisted by the intrinsic tendency of BN to form planar domains on the seed surface (as shown in Figure 8 b–d). This leads to a well‐defined cap with continuity with the BN island in one side (white arrows), while the other side rests perpendicularly on the seed surface (yellow arrows). The nanotube forms as a result of addition of B_x_N_y_H_z_ precursors at the contact points with the seed. The precursors continue to add on the seed surface forming BN facets extending from the BN island to the nanotube's perpendicular component connecting with it discontinuously, thereby terminating the nanotube growth. This mechanism accounts for the shell‐tube thickness mismatch and therefore suggests that the B seed acted as a scaffold while the BN island served as the BNNT nucleation site. This proposed pathway, while inferred from reproducible seed‐tube structures found in ex situ post‐synthesis TEM imaging, remains speculative in nature and to be verified in future in situ or molecular dynamics studies.

## 5 Conclusion

This work investigates a causal relationship between the growth of active boron droplets (B seeds) and BNNT selectivity in an inductively coupled plasma process. Overall, successful nanotube nucleation is more likely to take place on a B seed within the range of ∼5–50 nm. Several factors influenced the size and abundance of B droplets during their in‐flight growth, namely, B partial pressure, turbulence, and residence time. B partial pressure was controlled by adjusting the feedstock feed rate, while the turbulence and residence time in the droplet formation zone were controlled by adjusting the plasma power. Increasing the feed rates at a fixed power resulted in a constant B seed range (7–20 nm), but with varying abundances, which strongly correlated with the BNNT selectivity. Increasing the power at a fixed feed rate added a layer of complexity, as it affected both the size range and abundance of the seeds; the highest abundance again strongly correlated with higher BNNT selectivity. By simultaneously adjusting both operating conditions, it was possible to control these parameters effectively to improve BNNT selectivity at non‐optimal conditions. The reason for the change in the B seed size with the power increase was presumed to be the cooling rate during the BNNT nucleation step, as it affects the droplet size and the interaction of precursors on its surface. However, the nanotube diameter did not correlate strongly with the B seed diameter, possibly due to the hybrid growth mode that formed a golf club‐like structure. Moreover, the nanotube wall thickness was seen to not necessarily correlate with the thickness of the BN shell encapsulating the B seed, which was attributed to the growth of BN facets that terminated BNNT growth, consistent with the shell‐tube dual connectivity. Finally, we believe that this work provides insights for optimizing the synthesis conditions to improve BNNT selectivity and structural properties for targeted applications.

## Conflicts of Interest

The authors declare no conflicts of interest.

## Supporting information




**Supporting File**: smll71965‐sup‐0001‐SuppMat.pdf

## Data Availability

The data that support the findings of this study are available from the corresponding author upon reasonable request.
