# Supplementary material for: A Causal Relationship Between Boron Seeds and the Selectivity and Growth Mode of Boron Nitride Nanotubes in Inductively‐Coupled Plasma
Source: Small. 2026 Jan 7;22(9):e13512. doi: 10.1002/smll.202513512 (PMC12895144; doi:10.1002/smll.202513512)
Supplement: Supplementary file 1 — Supporting File: smll71965‐sup‐0001‐SuppMat.pdf [file SMLL-22-e13512-s001.pdf]

Supporting Information

A causal relationship between boron seeds and the selectivity and growth mode of boron nitride nanotubes in inductively-coupled plasma

A. Alrebh[1*], L. Gaburici[1], D. Ruth[1], M. Plunkett[1], C.T. Kingston[1*] and K. S. Kim[1,2*]

[1]Quantum and Nanotechnologies Research Centre, National Research Council, Ottawa, ON K1A 0R6, Canada

[2]Department of Mechanical and Industrial Engineering, University of Toronto, Toronto, Ontario M5S 3G8, Canada

[*]Corresponding authors: A. Alrebh (Aqeel.Alrebh@nrc-cnrc.gc.ca)

C.T. Kingston (Christopher.Kingston@nrc-cnrc.gc.ca)

K. S. Kim (KeunSu.Kim@nrc-cnrc.gc.ca)

Nucleation temperature and critical cluster size: The nucleation temperature of B was calculated based on the homogeneous nucleation theory and utilizing thermodynamic equilibrium data in Fig. S1. Then, it was used to determine the critical size of B clusters (d_{pcr}, nm), and the flux of B monomers onto the clusters surface (φ, m^{-2} s^{-1}). Then, the range between the B nucleation temperature and the BN formation temperature was used to identify the axial distance where B growth takes place in Fig. S2a. The gas velocity (Fig. S2b) in this axial distance was used to determine the average local residence time for B droplet growth $\bar{t}_l$.

The nucleation temperature, T (K), was calculated based on a model for homogeneous nucleation rate (J),[1] which assumed to be 10^6 m^{-3} s^{-1}:[2]

$$J = \frac{\beta_{ij}\, n_s^2\, S}{12} \sqrt{\frac{\Theta}{2\pi}} \cdot \exp\left[\Theta - \frac{4\,\Theta^3}{27\,[\ln(S)]^2}\right] \tag{S1}$$

Here, β_{ij} (m^3 s^{-1}) is the collision frequency function for i-mer and j-mer, given by eq. (S2), and n_s is the equilibrium concentration for a saturated B vapor (m^{-3}) at a given temperature given by $n_s = P_o/k_BT$, where k_B is the Boltzmann's constant (m^2 kg s^{-2} K^{-1}). S is the saturation ratio, and it must be greater than unity (S > 1) for nucleation to occur. It is given by $S = P_o/P_e$. The partial pressure of boron, P_o (Pa) was approximated based on the thermodynamic equilibrium data; and P_e (Pa) is the equilibrium vapor pressure which can be determined using eq. (S3)[3]. The dimensionless surface energy, Θ, is given by eq. (S4).

$$\beta_{ij} = \left(\frac{3\,v_1}{4\pi}\right)^{\frac{1}{6}} \sqrt{\frac{12\,k_BT}{\rho_p}\left(\frac{1}{i}+\frac{1}{j}\right)}\, \left(i^{1/3}+j^{1/3}\right)^2 \tag{S2}$$

$$P_e = 101325 \cdot \exp\left[13.95 - \frac{57745}{T}\right] \tag{S3}$$

$$\Theta = \frac{\sigma\, s_1}{k_{BT}} \tag{S4}$$

where σ (N m^{-1}) and s_1 (m^2) are, respectively, the surface tension and surface area of B monomer.

In eq. (S2), i and j are equal to 1 for collisions between monomers, and the monomer molar volume v_1 (m^3 $atom^{-1}$) can be found by:

$$v_1 = \frac{M_w}{\rho_p N_A} \quad (S5)$$

where M_w (kg mol^{-1}) is the molar mass of B, ρ_p (kg m^{-3}) is the particle mass density of B in liquid phase, and N_A is the Avogadro's number. Both ρ_p and σ were determined using the following empirical formulas,[4] which are applicable in the 2300-3300 K range:

$$\sigma = 1.178 - 3.8 \times 10^{-5}\ T \quad (S6)$$

$$\rho = 2468 - 5.31 \times 10^{-2}\ T \quad (S7)$$

In eq (S4), s_1 can be found from:

$$s_1 = 4\pi \left(\frac{3\ v_1}{4\pi}\right)^{\frac{2}{3}} \quad (S8)$$

B nuclei critical size (d_{pcr}, m) can be estimated using eq. (S9),[5] and monomer flux (φ, m^{-2} s^{-1}) using eq. (S10):[6]

$$d_{pcr} = \frac{4\ \sigma\ v_1}{k_B\ T\ \ln(S)} \quad (S9)$$

$$\varphi = \left(\frac{k_B T}{2\pi\ \rho_p v_1}\right)^{1/2} n_s \quad (S10)$$

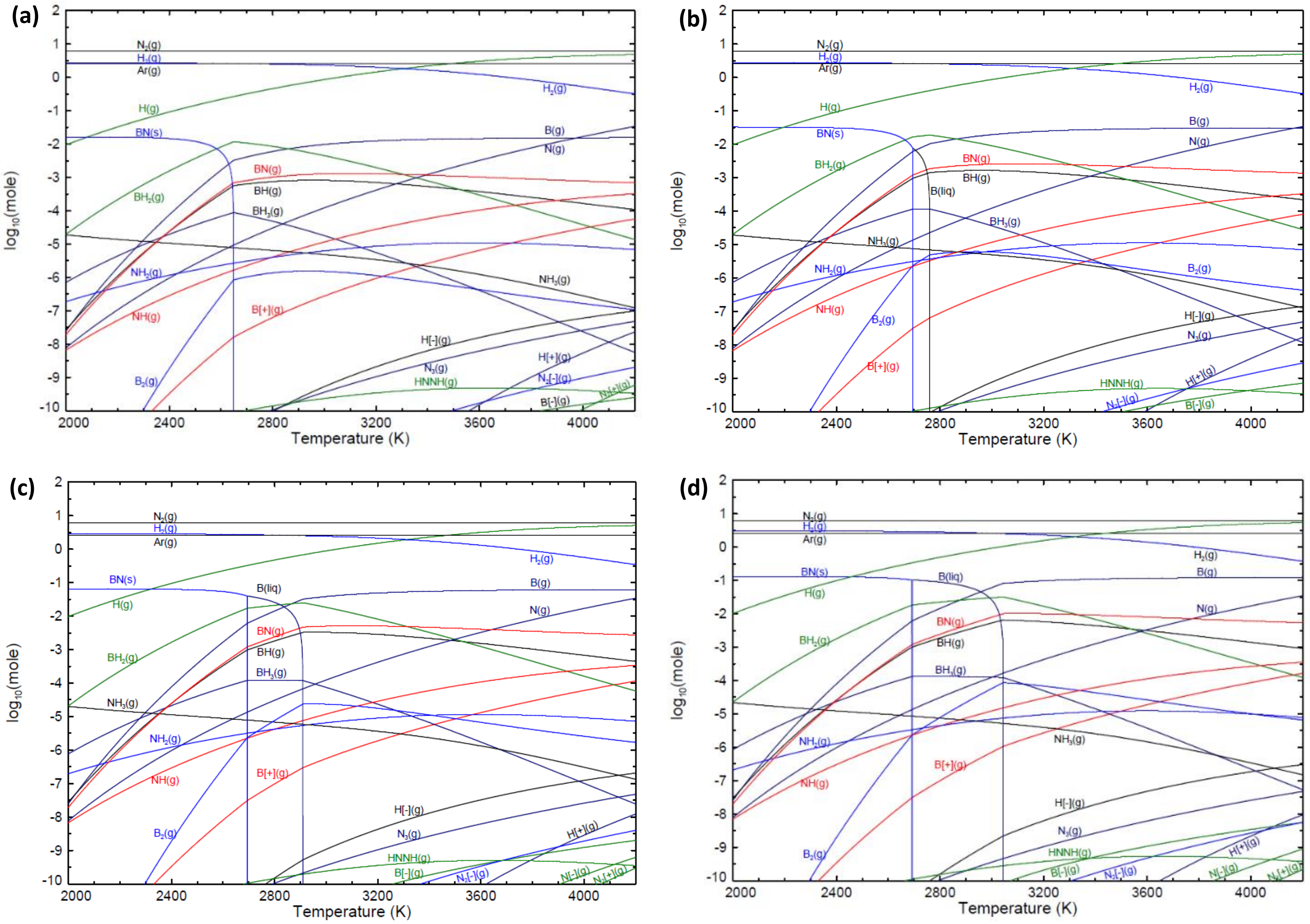

Figure S1: Thermodynamic equilibrium for systems shown in Table 1 for AB feed rates of a) 0.5, b) 1, c) 2, and d) 4 g/min. The results and graphs were generated using FactSage^{TM} 8.2 software. B mole fractions were used to calculate the B nucleation temperatures.

Residence time and cooling rate: Utilizing Fig. S2, the residence time ($\bar{t} = \Delta z/\bar{u}$, s) in the system was estimated by summing $\bar{t}$ for each computational node along the entire length of the plasma torch and the reaction column, where Δz is the length of a computational cell (m) along the axis of symmetry, and $\bar{u}$ is the average velocity (m s^{-1}) in the cell. The summation was done over the entire system to find the total residence time, $\bar{t}_t$. The local average residence time, $\bar{t}_l$, for the B droplet formation zone was evaluated by summing $\bar{t}$ for all cells in the axial distance covering the range from the B nucleation to the BN formation temperatures. The average cooling rate (K s^{-1}) in this zone was estimated using $\Delta T/\Delta t = -\Delta T/\Delta z \cdot \bar{u}$.

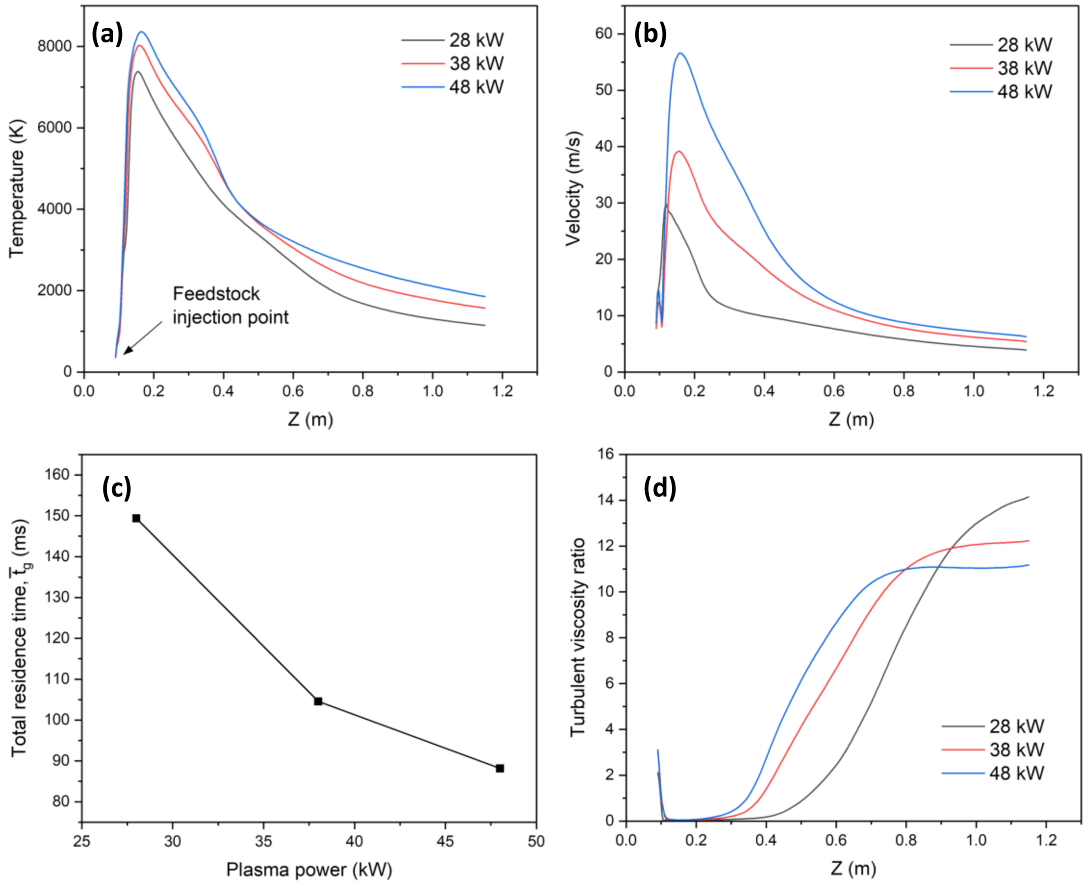

Figure S2: CFD simulation results for (a) temperature, (b) velocity, (c) total residence time, and (d) turbulent to laminar viscosity ratio profiles of hot gas in the plasma torch and the reaction column along the axis of symmetry (z-axis). These profiles were derived by extrapolation/interpolation of data presented in reference 7 for the same geometry and process conditions but at different plasma powers. The feedstock was not considered in the calculations.

Discussion on TG/DTG analysis: The analysis of the off-gas released from the TGA (Fig. 2f) and DTG (Fig. S3a) is discussed here utilizing FTIR spectra obtained by the FTIR module coupled with the TGA instrument. As the temperature increases, adsorbed water and ammonia first desorb off the surface of particles (Fig. S3b and c, respectively). Then, B particles start to oxidize above 600 °C with an increasing rate until 750 °C where the oxidation declines. At this stage, no gases were detected other than carbon dioxide likely due to impurities (Fig. S3d). Here, the oxide layer coating the particles minimizes further oxidation due to having extremely low diffusion for oxygen. Low crystalline thin BN nanosheets, *a*BN, *t*BN and possibly defective BNNTs then start to decompose releasing nitrogen which reacts with oxygen from nitrous oxide (Fig. S3e), and boron which undergoes oxidation. Transmission Electron Microscopy (TEM) image in Fig. S3f, and Electron Energy Loss Spectroscopy (EELS) maps for boron and nitrogen are shown in Fig. S3g and h representing a direct structural characterization confirming that B particles are encapsulated with BN shells.

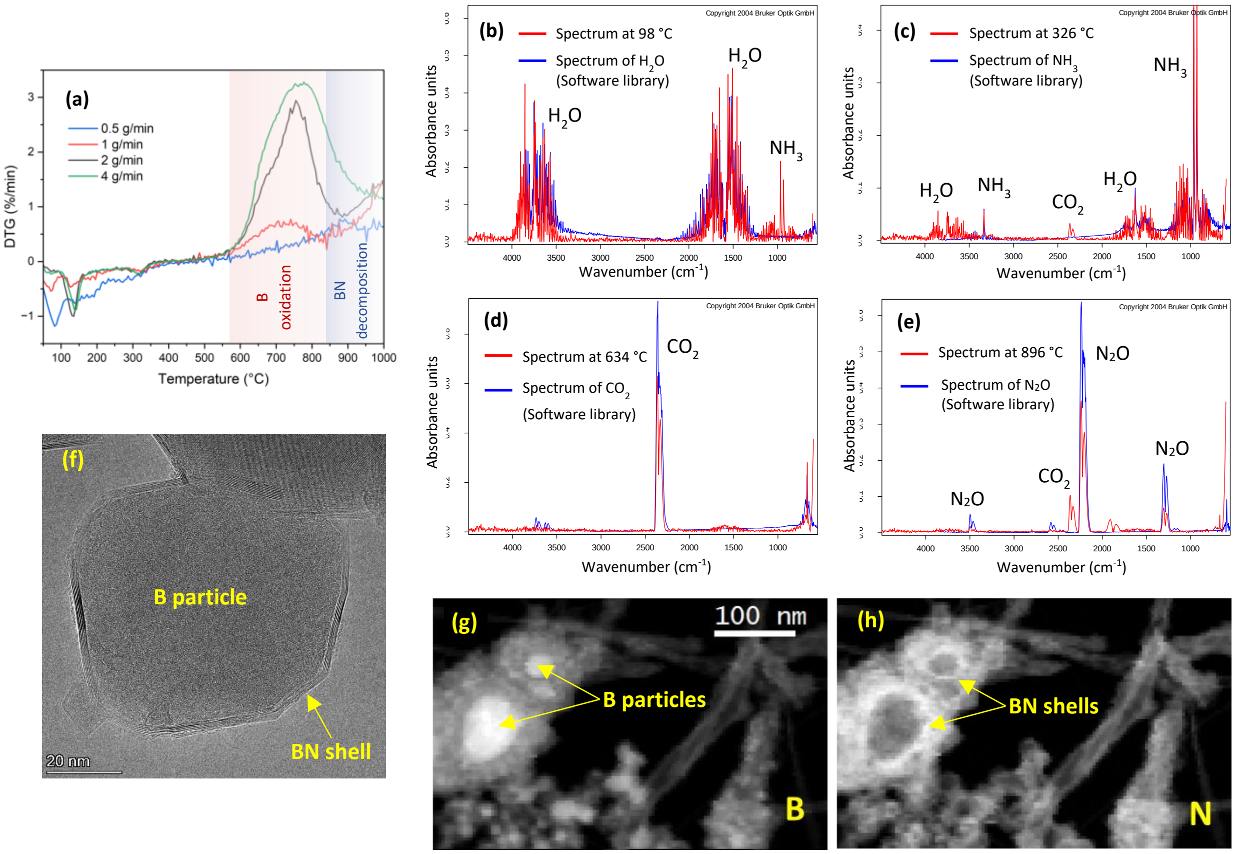

Figure S3: (a) DTG graph corresponding to TGA shown in Fig. 2f. Typical IR spectra of off-gases acquired during the TGA analysis for BNNTs showing (b) H_2O at 98 ºC, (c) NH_3 at 326 ºC, (d) CO_2 at 634 ºC and (e) N_2O at 896 ºC. The IR spectra in red represent experimental data for the material obtained at 1 g/min and the blue ones belong to the indicated species as found in the instrument software library (Library name: EPA-NIST.S01). These panels are screencaps from the software. (f) TEM image for BN-encapsulated B particle. (g) and (h) EELS elemental maps showing residual B particles coated with BN shells[8]. The B signal dominates the particle core in (arrows in g), while N is enriched in the surrounding shell region (arrows in h). Panels (g) and (h) are adapted with permission from Ref. 8 © 2023 Elsevier.

Curve fitting for B particle/seed distributions: The equation and parameters obtained by fitting the lognormal distribution curves for B particle and seed distributions in OriginLab are in eq. (S12) and Table S1.

$$f(x) = \frac{a}{b\sqrt{2\pi}\,x}\exp\left[-\frac{\ln^2\left(\frac{x}{c}\right)}{2b^2}\right] \quad (S12)$$

Table S1: Parameters of lognormal distributions used in eq. (S12) for B particles and B seeds at various process conditions

Process conditions	B particles			B seeds		
	**a**	**b**	**c**	**a**	**b**	**c**
0.5 g/min, 38 kW	1450	0.70036	14.03616	120	0.46464	13.65202
1 g/min, 38 kW	1450	0.69553	17.74907	120	0.52546	15.23556
2 g/min, 38 kW	1450	0.73286	26.78951	120	0.54121	15.67950
4 g/min, 38 kW	1450	0.73106	48.50629	120	0.41032	11.76553
1 g/min, 28 kW	1450	0.74246	22.16659	120	0.39361	8.43315
1 g/min, 48 kW	1450	0.98732	37.90282	120	0.50785	19.74693

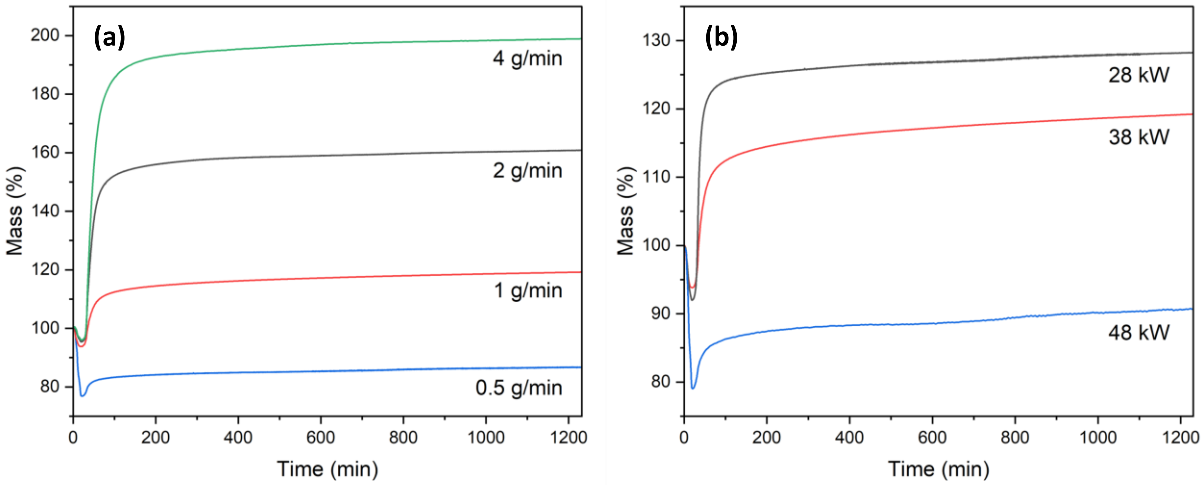

Figure S4: (a) Thermograms of as-produced materials synthesized at various (a) ammonia borane feed rates, and (b) plasma powers. The panels show the mass percentage change as a function of time. Materials were heated from room temperature to 700 °C at 20 °C/min and then held for 20 h.

Table S2: Estimated relative B seed relative abundance for BNNT samples obtained at various AB feed rates and plasma powers. Residual B ratios were obtained from Fig. S4.

AB feed rate (g/min)	Relative B seed abundance (count)
0.5	35
1	85
2	130
4	70

Plasma power (kW)	Relative B seed abundance (count)
28	30
38	85
48	5
-	-

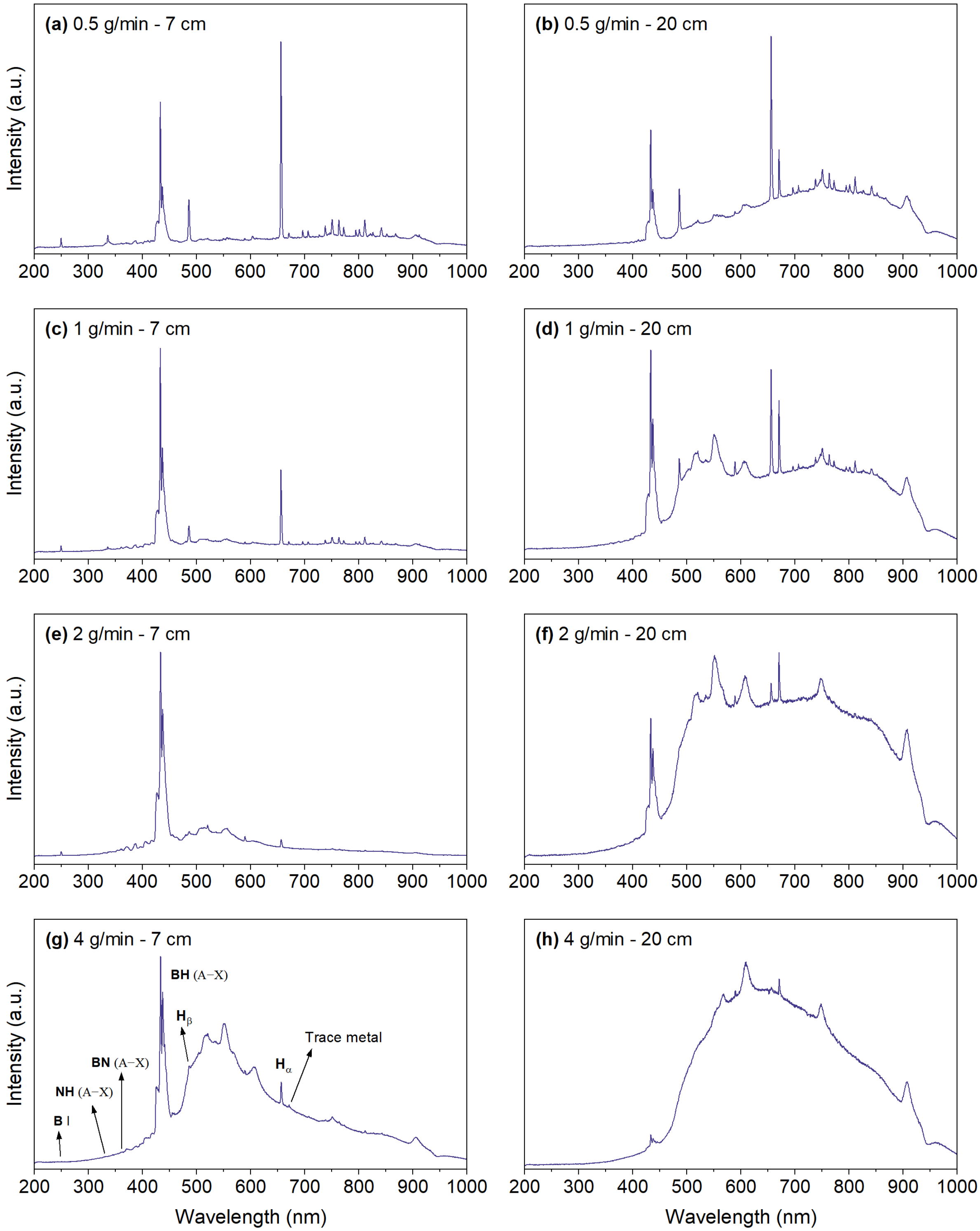

Figure S5: Representative OES spectra obtained for various ammonia borane feed rates at 7 and 20 cm below the torch nozzle, used to construct Fig. 4c and d. Peak assignment is shown in (g).

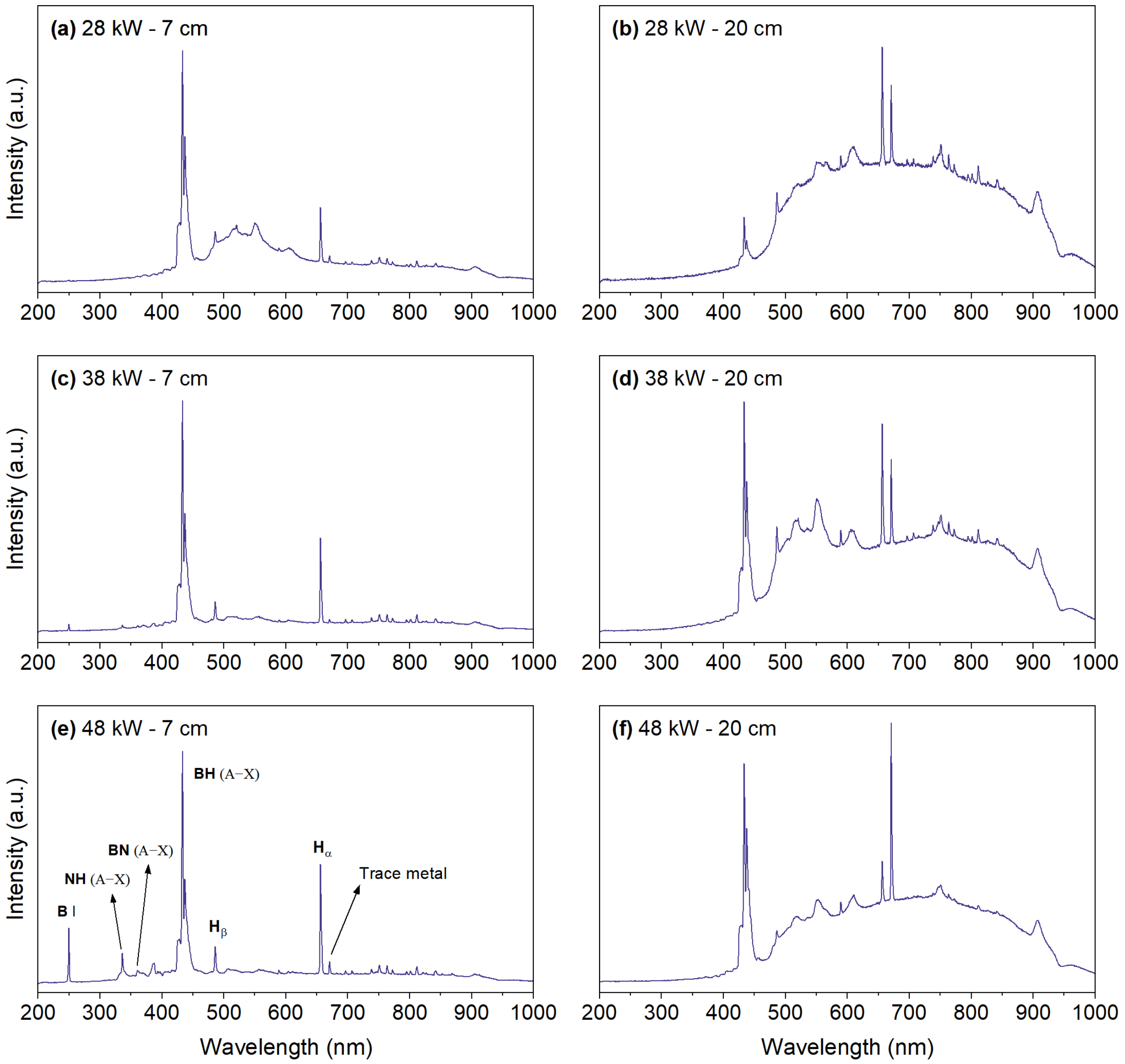

Figure S6: Representative OES spectra obtained various powers at 7 and 20 cm below the torch nozzle, used to construct Fig. 7b and c. Peak assignment is shown in (e).

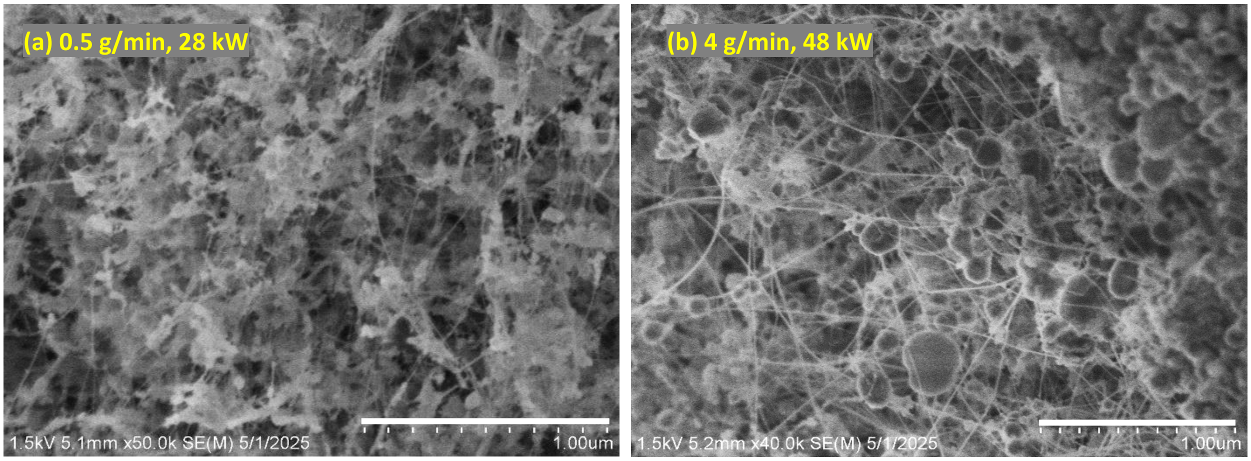

Figure S7: SEM images of BNNTs synthesized at (a) low feed rate (0.5 g/min) and low power (28 kW), and (b) high feed rate (4 g/min) and high power (48 kW). Scale bar 1 μm. Compared to Fig. 2a and d (or Fig. 5a and c), respectively, these conditions show a notable improvement in BNNT selectivity, demonstrating that tuning the seed size to better overlap with the B droplets size can increase the seed abundance.

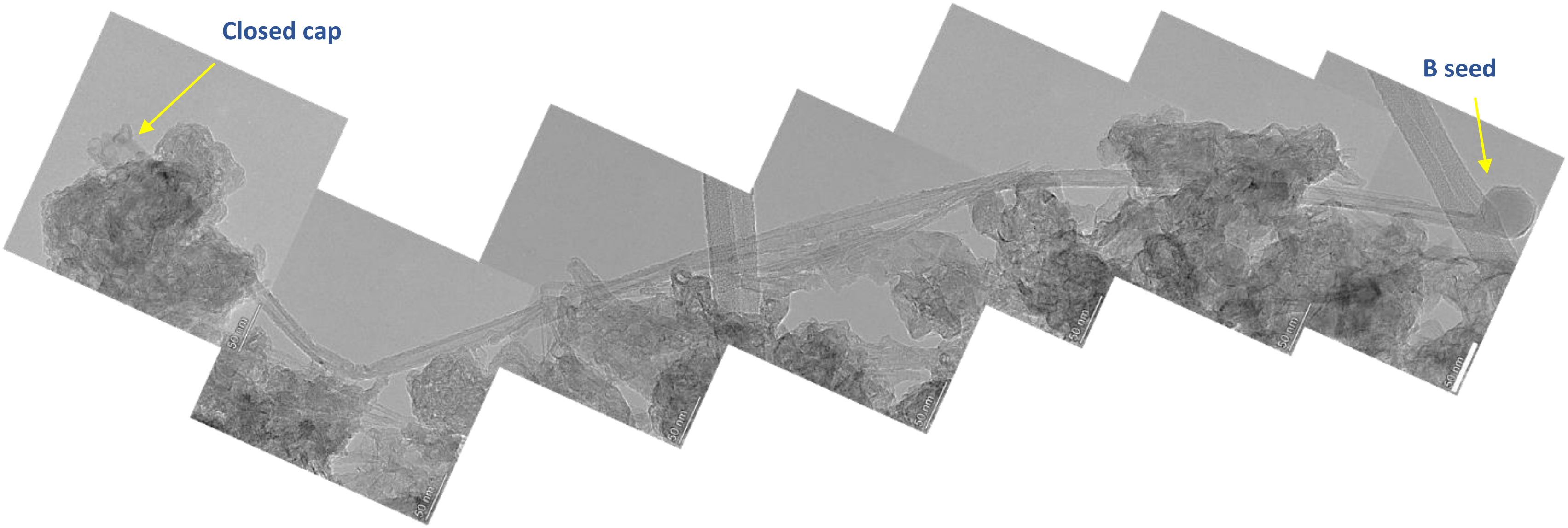

Figure S8: A group of TEM images demonstrating a nanotube with one end having a B seed (right) and the other end have a closed cap (left). The scale bar is 50 nm.

References:

(1) Girshick, S.; Chiu, C.-P.; McMurry, P. Time-dependent aerosol models and homogeneous nucleation rates. *Aerosol science and technology* **1990**, *13* (4), 465-477. DOI: https://doi.org/10.1080/02786829008959461.

(2) Kousaka, Y.; Nomura, T.; Alonso, M. Simple model of particle formation by homogeneous and heterogeneous nucleation. *Advanced Powder Technology* **2001**, *12* (3), 291-309. DOI: https://doi.org/10.1163/156855201750537866.

(3) Chen, B.; Xia, Z.; Huang, L.; Hu, J. Ignition and combustion model of a single boron particle. *Fuel Processing Technology* **2017**, *165*, 34-43. DOI: https://doi.org/10.1016/j.fuproc.2017.05.008.

(4) Millot, F.; Rifflet, J.-C.; Sarou-Kanian, V.; Wille, G. High-temperature properties of liquid boron from contactless techniques. *International journal of Thermophysics* **2002**, *23* (5), 1185-1195. DOI: https://doi.org/10.1023/A:1019836102776.

(5) Shigeta, M.; Watanabe, T. Numerical analysis for co-condensation processes in silicide nanoparticle synthesis using induction thermal plasmas at atmospheric pressure conditions. *Journal of materials research* **2005**, *20* (10), 2801-2811. DOI: https://doi.org/10.1557/JMR.2005.0351.

(6) Girshick, S. L.; Chiu, C. P. Kinetic nucleation theory: A new expression for the rate of homogeneous nucleation from an ideal supersaturated vapor. *The journal of chemical physics* **1990**, *93* (2), 1273-1277. DOI: https://doi.org/10.1063/1.459191.

(7) Kim, K. S.; Sigouin, G.; Cho, H.; Couillard, M.; Gallerneault, M.; Moon, S. Y.; Lee, H. S.; Kim, M. J.; Jang, S. G.; Shin, H. Insight into BN impurity formation during boron nitride nanotube synthesis by high-temperature plasma. *ACS omega* **2021**, *6* (41), 27418-27429. DOI: https://doi.org/10.1021/acsomega.1c04361.

(8) Alrebh, A.; Ruth, D.; Plunkett, M.; Gaburici, L.; Couillard, M.; Lacelle, T.; Kingston, C.; Kim, K. Boron nitride nanotubes synthesis from ammonia borane by an inductively coupled plasma. *Chemical Engineering Journal* **2023**, *472*, 144891. DOI: https://doi.org/10.1016/j.cej.2023.144891.
